# Multidrug Resistant Enteric Bacteria from Cancer Patients Admitted in Douala Laquintinie Hospital, Littoral Region of Cameroon

**DOI:** 10.1155/2024/2084884

**Published:** 2024-07-12

**Authors:** Michael F. Kengne, Ornella D. Tsobeng, Ballue S. T. Dadjo, Victor Kuete, Armelle T. Mbaveng

**Affiliations:** Department of Biochemistry Faculty of Science University of Dschang, Dschang, Cameroon

## Abstract

Patients with cancer have weakened immune systems, making them more vulnerable to infections. This study was carried out to determine the bacterial origins of enteric disorders in cancer patients and noncancer patients at the Oncology Department of Laquintinie Hospital in Douala. A cross-sectional study was conducted from October 2021 to March 2023. Stool samples from 307 cancer patients with enteric disorders and 200 noncancer patients with enteric disorders were examined to diagnose the presence of bacteria using various techniques. Among all participants in this study, 62.13% were female and 37.87% were male. The average age of the participants was 46.38 ± 15.81 years, with a minimum age of 10 years and a maximum age of 84 years. The average age of participants was significantly higher (*p* < 0.000) in cancer patients (49.54 ± 14.65 years) compared to noncancer patients (41.53 ± 16.33 years). *Proteus mirabilis, Proteus vulgaris, Salmonella typhi, Enterobacter cloacae, Klebsiella pneumoniae, Yersinia intemedia, and Klebsiella oxytoca* were more frequently isolated in cancer patients than in noncancer patients, with the respective percentages of 56.25% *versus* 43.75%, 50.00% *versus* 50.00%, 61.66% *versus* 38.34%, 66.66% *versus* 33.34%, 72.22% *versus* 27.78%, 80.00 *versus* 20.00%, and 100% *versus* 0.00%. Most isolates were sensitive to imipenem (IMP), gentamicin (GEN), and amikacin (AMK). *Proteus vulgaris,* the most prevalent isolate, showed significantly high resistance (with *p* < 0.05) in cancer patients compared to noncancer patients at amoxicillin/clavuranic acid (AMC) (89.13% *versus* 41.30%), ceftriaxone (CTR) (63.04% *versus* 39.13%), ciprofloxacin (CIP) (65.22% *versus* 34.18%), and tetracycline (TET) (93.48% *versus* 63.04%). Multidrug resistance was observed in cancer patients compared to noncancer patients for *Klebsiella pneumoniae* (85.00% *versus* 60.00%), *Salmonella typhi* (84.62% *versus* 60.00%), and *Klebsiella oxytoca* (86.49% *versus* 43.48%). The increase in the number of Gram-negative infections among cancer patients, as shown in the present study, highlights the need for broad-spectrum therapy and effective planning of control programs to reduce bacterial diseases among cancer patients.

## 1. Introduction

Cancer is one of the leading causes of death worldwide, particularly in developing countries. Cancers of the lung, liver, stomach, colon, breast, and leukemia are frequently fatal [1]. Cancer patients have an increased risk of infectious complications, mainly of bacterial origin [2]. Infections are one of the most serious complications and the leading cause of morbidity and mortality in cancer patients [3]. Cancer patients are immunocompromised due to a primary or secondary immunodeficiency disorder or the use of anticancer agents that depress one or more components of the immune system [4, 5]. An immunocompromised host presents an impairment of phagocytic, cellular, or humoral immunity, which increases the risk of infectious complications or serious opportunistic processes [6, 7]. It is also considered that cancer patients' bacterial infections arise from a reduction of their cell-mediated immunity as a result of receiving anticancer treatment repeatedly and continuously [8]. Anticancer treatment has also been shown to play an important role in the spread of infectious events in cancer patients [9]. Several bacteria, such as *Klebsiella pneumoniae*, *Pseudomonas aeruginosa,* and *Escherichia coli,* can cause infections in cancer patients [10]. Loss of antibiotic effectiveness due to bacterial resistance to antibiotics poses an urgent threat to the continued success of cancer treatment, which is often responsible for complications that can lead to the early death of patients [11]. There is a reduction in bacterial sensitivity to almost all available antibiotics [12]. In cancer patients, anticancer treatment promotes the evolution of the microbiota and the production of antibiotic-resistant mutants from intestine bacterial commensals [13]. Several cytotoxic drugs used in cancer chemotherapy have antimicrobial properties and have a direct effect on increasing bacterial mutation rates, mainly through activation of the SOS response and horizontal gene transfer [13–19]. This has an impact on the contribution to the diffusion of antibiotic resistance mechanisms in bacterial populations in response to stress leading to serious clinical consequences [20–24]. Cancer patients are treated with substances that can modify bacterial DNA and increase the rate of bacterial mutation, resulting in antibiotic resistance [13–19]. Infections with antibiotic-resistant pathogens in the general population and in cancer patients are one of the leading causes of mortality and constitute a public health problem. The evolution of bacterial resistance requires up-to-date knowledge of the rate of resistance as well as an understanding of the risk factors involved in this resistance. To our knowledge, in Africa in general and Cameroon in particular, there are no studies involving cancer patients undergoing anticancer chemotherapy or not suffering from enteric infections. The objective of this work was to study the epidemiological profiles of pathogenic bacteria from the enteric tract of cancer patients in the city of Douala, Littoral Region of Cameroon, while determining the influence of anticancer treatment on the extent of antibiotic resistance in cancer patients.

## 2. Material and Methods

### 2.1. Study Design

This was a cross-sectional study carried out from October 2021 to March 2023 at the Laquintinie hospital in Douala located in the Littoral Region of Cameroon. It is one of the reference centers that houses an oncology department in the city of Douala.

### 2.2. Study Population

This study included cancer patients and noncancer patients suffering from enteric disorders who came for consultation. Five hundred and seven (507) individuals were willing to voluntarily participate in this study. They consisted of 307 cancer patients and 200 noncancer patients. Patients whose doctors ordered stool examinations and who had not received any specific antibacterial treatment in the previous two weeks were included in this study. Pregnant women, diabetic patients, HIV-positive patients, burn victims, and patients taking estrogens were not included in this study.

### 2.3. Ethical Approval

Ethical authorizations were obtained from the National Research Ethics Committee for Human Science (CNERSH), Yaoundé-Cameroon, that delivered an ethical clearance (with reference number No 2022/09/129/CE/CNERSH/SP) and from the institutional ethics committee of the University of Douala (CIE-UD) (with reference number No 3127/CEI-UDo/06/2022/T).

### 2.4. Collections of Stool Samples

Stool samples were collected in sterile containers, taking all precautions to avoid contamination.

### 2.5. Isolation and Identification of Bacteria

A single stool sample was collected from each study participant using a sterile, disinfectant-free container. Stool samples were quickly transported to the laboratory and grown on *Salmonella-Shigella* (SS) agar, Hektoen enteric agar, eosin-methylene blue (EMB) agar, and MacConkey agar, are selective and differential culture media used for the isolation of Gram-negative bacilli, including bacteria of the Enterobacteriaceae family, which are often responsible for enteric disorders. We chose these culture media because they are readily available and allow us to isolate and suspect bacteria easily, as follows: *Salmonella/Shigella* medium was used, on which *Salmonella* form colorless colonies with or without a black center (lactose negative and H_2_S positive). This allowed us to suspect *Salmonella* bacteria. EMB and Hektoen agars were used simultaneously to isolate *Klebsiella* spp. because the pink colonies with a purple center on EMB mucosal bulge 5 mm in diameter with no metallic sheen raised the suspicion of *Klebsiella* spp., and these pink colonies had a yellow coloration on Hektoen agar. The bluish appearance of the colonies pointed towards *Enterobacter* spp. on EMB medium, and this bluish colony was then inoculated onto Hektoen agar, where, after 24 hours' incubation at 37°C, yellow colonies were observed, indicating that *Enterobacter* spp. were present. Bacteria of the Proteus genus are yellow or salmon-colored with a black center on Hektoen medium and greyish with a film around the colony when grown on EMB agar. Isolated bacteria were identified on the basis of colonial morphology and Gram stain, and using API 20E galleries (Biomérieux, Lyon, France) as described by the manufacturer.

### 2.6. Antibiotic Susceptibility Testing

Antimicrobial susceptibility testing was carried out on all isolates using the diffusion method as described by Kirby-Bauer on Mueller-Hinton agar (TITAN BIOTECH LTD, Rajasthan, India), in accordance with the recommendations of the European Committee on Antimicrobial Susceptibility Testing (EUCAST) [25, 26]. The antibiotics tested included amikacin (AMK, 30 *μ*g), amoxicillin (AMX, 10 *μ*g), amoxicillin/clavuranic acid (AMC, 20/10 *μ*g), aztreonam (ATM, 30 *μ*g), ceftazidime (CAZ, 30 *μ*g), ceftriaxone (CTR, 30 *μ*g), cefuroxin (CXM, 30 *μ*g), cefoxitin (FOX) (10 *μ*g); cefotaxime (CTX, 10 *μ*g), colistin (COL, 30 *μ*g), ciprofloxacin (10 *μ*g), erythromycin (ERY, 10 *μ*g), fosfomycin (FOS, 10 *μ*g), gentamicin (GEN, 10 *μ*g), imipenem (IMP, 10 *μ*g), naxidilic acid (NAL, 10 *μ*g); nitrofurantoin (NIT, 100 *μ*g), ofloxacin (OFX, 5 *μ*g), piperacillin (PRL, 10 *μ*g), tetracycline (TET, 30 *μ*g), trimethoprim-sulfamethoxazole (COT, 10 *μ*g), and vancomycin (VAN, 10 *μ*g). They were purchased from Singapore Bioscience PTE Ltd, Singapore.

Briefly, the test isolate was emulsified in peptone until turbidity was like that of the 0.5 McFarland standard. A sterile cotton swab was dipped into the suspension and dabbed evenly over the entire surface of the agar plate to obtain semi-confluent growth. The zones of inhibition around the antibiotic disks were measured and evaluated upon incubation using the Clinical and European Committee on Antimicrobial Susceptibility Testing (EUCAST) breakpoint criteria [26, 27]. Quality control of antibiotic disks (Oxoid, UK), media (Accumix, Mol, Belgium), and incubation conditions was ensured using *Escherichia coli* ATCC 25922. Isolates showing resistance to three categories or more of antibiotics have been considered multiresistant bacteria [28].

### 2.7. Statistical Analyses

All data were stored in a common database and statistically analyzed using Epi Info™ version 7.2.2.6 (CDC, 1600 Clifton Road, Atlanta, GA 303294027, USA). For categorical data, chi square was used to quantify significant differences between cancer and noncancer patients. To investigate the relationship between the rate of isolation of bacterial agents and cancer status, the chi-square test and Fisher's exact test were used to compare proportions. A *p*-value of <0.05 was considered statistically significant. Crude ORs and exact 95% CIs were calculated to assess the potential relationship between prior antibiotic exposure and multidrug resistance in bacteria. Bivariate logistic regression was used to assess whether prior antibiotic exposure was a risk factor for multidrug-resistant bacteria.

## 3. Results

### 3.1. Sociodemographic Characteristics of the Study Population and Distribution of Different Types of Cancer

Of the five hundred and seven cancer and noncancer patients with enteric disorders recruited, 192 (37.86%) were males and 315 (62.14%) were females. Among men, 109 (56.77%) were cancerous and 83 (43.23) were noncancerous. Among the women, 198 (62.85%) suffered from cancer, and 117 (37.15%) did not suffer from cancer. There was a significant difference in the distribution of patients in different age groups. The maximum number of cancer patients in the age group above ≥60 years was 73.91%, followed by 69.66% and 67.14% in the age groups of [50–60] years and [40–50] years, respectively. The mean age of our study participants was significantly higher in cancer patients (49.54 ± 14.65 years) than in noncancer patients (41.53 ± 16.33 years), and the age ranged between 10 and 82 years old (Table 1).

**Figure 1:**
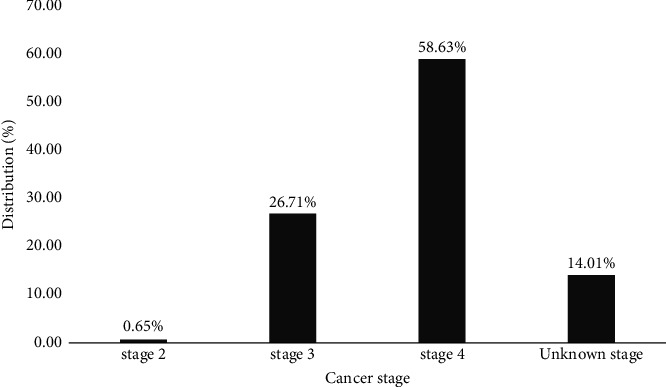
Distribution of different stages of the disease in the study population.

Three hundred and seven cancer patients were enrolled in this study, with 98 having breast cancer (31.91%) and 42 having cervical cancer (13.68%). Esophageal cancer was the least represented, with 5 cases. Among the patients suffering from cancer, 188 (61.24%) were undergoing anticancer chemotherapy, compared to 119 (38.76%) who had not undergone anticancer chemotherapy (Table 2). In this study, we used the extension work-up prescribed by the oncologist to determine the stage of the cancer according to a clinical classification system by the TNM classification [29].  Figure 1 shows that 180 patients suffering from cancer had a level of tumor progression at stage 4, that is, a frequency of 58.63%, followed by stage 3 with 82 patients with a percentage of 26.71 and stage 2 being less represented with a percentage of 0.65.

### 3.2. Prevalence of Enterobacteria Responsible for Infections in Study Groups

**Figure 2:**
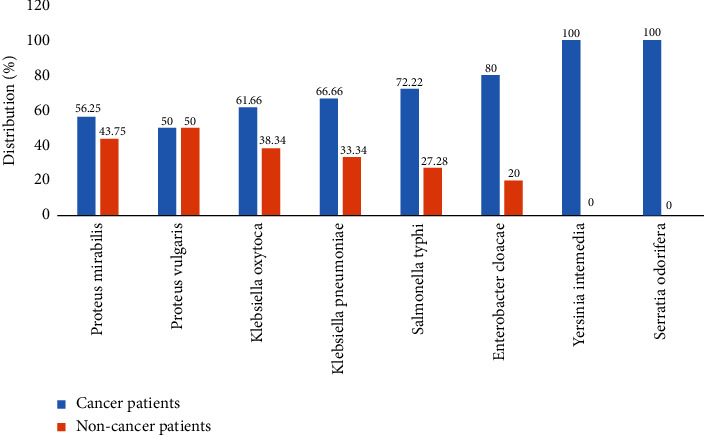
Distribution of intestinal pathogenic bacteria among patients with enteric disorders enrolled in the study.

A total of 150 bacteria were isolated from cancer patients (48.85%) and 100 from noncancer patients (50%).  Figure 2 shows that *Proteus mirabilis, Klebsiella oxytoca, Klebsiella pneumoniae, Salmonella typhi, Enterobacter cloacae,* and *Yersinia intemedia* were isolated more frequently in the stool samples of cancer patients than in noncancer patients, of with a percentage of 18 (56.43%), 37 (61.66%), 20 (66.66%), 13 (72.22%), 8 (80%), and 5 (100), respectively, compared to noncancer patients with values of 14 (43.75%), 23 (38.34%), 10 (33.34%), 5 (27.28%), 2 (20%), and 0 (0.00), respectively.

### 3.3. Resistance Risk Factors

Table 3 shows the different risk factors for resistance in the groups studied. It can be seen that the number of cancer patients with previous exposure to antibiotics (*n* = 203) was significantly higher (*p* < 0.001) than the number of noncancer patients (*n* = 56). Of the 307 cancer patients recruited in this study, 180 (58.63%) were hospitalized and 127 (41.37%) were recruited either in consultation or in the oncology department. The 200 noncancer patients were recruited mainly from outpatient departments. It should also be noted that 104 (55.32%) of the cancer patients had already received more than five courses of chemotherapy, compared with 84 (44.58%) who had received less than four courses of chemotherapy.

### 3.4. Antibiotic Resistance Profiles of *Proteus mirabilis* and *Proteus vulgaris*

The susceptibility of *Proteus mirabilis* and *Proteus vulgaris* isolates obtained from 22 different antibiotics was evaluated in this study.  Table 4 shows the susceptibility of the isolates to these antibiotics. *Proteus mirabilis* isolates showed high resistance rates in cancer patients compared to noncancer patients at AMC (72.22% versus 35.71%), PRL (88.89% versus 85.71%), CAZ (66.67% versus 35.71%), CIP (55.56% versus 42.86%), NAL (66.67% versus 35.71%), COT (72.22% versus 28.57%), and TET (88.89% versus 57.14%). Furthermore, the isolates of *Proteus vulgaris* presented rates of resistance significantly elevated in patients suffering from cancer compared to patients not suffering from cancer: AMX (91.30% versus 76.09%), AMC (89.13% versus 41.30%), PRL (100% versus 65.22%), CTR (63.04% versus 39.13%), CIP (65.22% versus 34.18%), COL (69.57% versus 30.43%), ATM (54.35% versus 23.91%), and TET (93.48% versus 63.04%).

### 3.5. Antibiotic Resistance Profile of Other Bacteria

All these bacteria had a sensitivity to IMP between 92.86% and 100%. Most isolates in our study were susceptible to IPM, GEN, and AMK. *Klebsiella oxytoca* showed significantly higher rates of resistance in cancer patients than in noncancer patients to AMC (62.16% versus 30.43%), CTX (67.57% versus 34.78%), CAZ (75.68% versus 17.39%), COL (54.05% versus 34.78%), TET (83.78% versus 56.52%), and NIT (51.35% versus 13.04%). *Klebsiella pneumoniae* isolates showed high resistance rates in cancer patients compared to noncancer patients at CTR (75.00% versus 30.00%), at CAZ (85.00% versus 40.00%), the COL (60.00% versus 10.00%), the TET (85.00% versus 40.00%), and the CXM (100% versus 30.00%). Furthermore, *Salmonella typhi* isolates presented high resistance rates in cancer patients compared to noncancer patients at COL (69.23% versus 0.00%), NIT (76.92% versus 0.00%), ATM (53.82% versus 20.00%), and CXM (100% versus 60.00%) (Table 5).

### 3.6. Multidrug-Resistant (MDR) Bacteria Isolated from Cancer and Noncancer Patients

**Figure 3:**
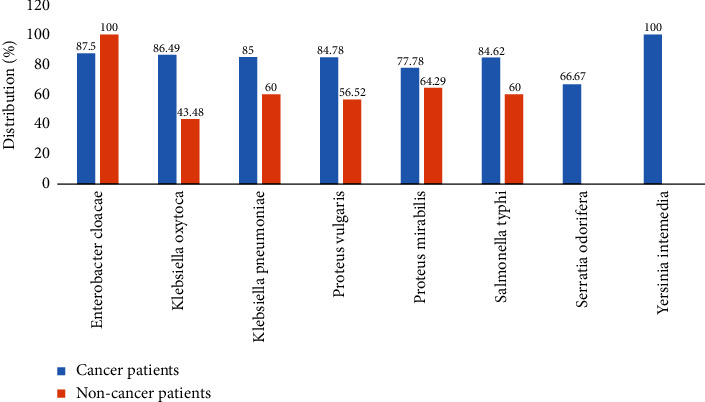
Frequency of appearance of multidrug-resistant (MDR) bacteria isolated from cancer and noncancer patients.

*Klebsiella pneumoniae* (85.00%), *Proteus mirabilis* (77.78%), *Salmonella typhi* (84.62%), *Klebsiella oxytoca* (86.49%), and *Proteus vulgaris* (84.78%) showed multidrug resistance to cancer patients, while Enterobacter cloacae (100%) showed multidrug resistance to noncancer patients (Figure 3).

### 3.7. Association of Prior Exposure to Antibiotic Therapy with Multidrug Resistance of *Proteus vulgaris*

Table 6 shows the association between prior antibiotic exposure and multidrug resistance of *P. vulgaris* in cancer and noncancer patients. It was found that cancer patients who had been exposed to prior antibiotic therapy were more likely to have a MDR profile of *P. vulgaris* than cancer patients who had not been previously exposed (OR = 2.33; 95% CI: 0.410-13.258; *p*=0.296; Table 6). This table also shows that noncancer patients who had been exposed to prior antibiotic therapy were slightly more likely to have an elevated *P. vulgaris* multidrug resistance profile than noncancer patients who had not been previously exposed (OR = 1.33; 95% CI: 0.359–4.945; *p*=0.462; Table 6). No statistically significant association was found between previous exposure to antibiotic therapy and the risk of multidrug resistance of *P. vulgaris* (*p* > 0.05).

### 3.8. Association of Prior Exposure to Antibiotic Therapy with Multidrug Resistance of *Klebsiella oxytoca*

Table 7 shows the association between prior antibiotic exposure and multidrug resistance of *Klebsiella oxytoca* in cancer and noncancer patients. It was found that no statistically significant association was found between prior antibiotic exposure and the risk of multidrug resistance of *Klebsiella oxytoca* (*p* > 0.05). Cancer patients who had been exposed to antibiotic therapy had a 1.70-fold higher risk of developing multidrug resistant *Klebsiella oxytoca* than noncancer patients, who had only a 1.11-fold risk.

### 3.9. Association of Prior Exposure to Antibiotic Therapy with Multidrug Resistance of *Klebsiella pneumoniae*

Table 8 shows the association between prior antibiotic exposure and multidrug resistance of *Klebsiella pneumoniae* in cancer and noncancer patients. It was found that no statistically significant association was found between prior exposure to antibiotic therapy and the risk of multidrug resistance in *Klebsiella pneumoniae* (*p* > 0.05).

### 3.10. Multidrug-Resistant (MDR) Bacteria Isolated from Cancer Patients According to Anticancer Treatment

**Figure 4:**
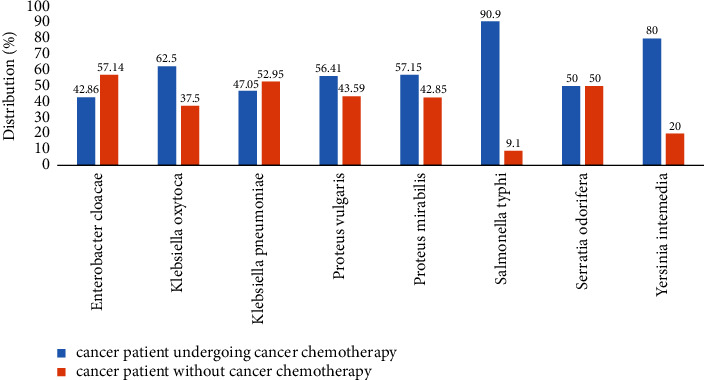
Frequency of appearance of multidrug-resistant (MDR) bacteria isolated from cancer patients according to anticancer treatment.

*Proteus vulgaris* (56.41%), *Salmonella typhi* (90.90%), *Klebsiella oxytoca* (62.50%), and *Proteus mirabilis* (57.15%) showed high multidrug resistance in cancer patients under cancer chemotherapeutic treatment, while Enterobacter cloacae (57.14%) and *Klebsiella pneumoniae* (52.95%) showed high multidrug resistance in cancer patients who did not undergo anticancer chemotherapeutic treatment (Figure 4).

## 4. Discussion

Cancer patients have a weakened immune system, making them more vulnerable to infection. With this in mind, we conducted a cross-sectional study to determine the bacterial origins of enteric disorders and to study the epidemiological profiles of pathogenic bacteria of the enteric tract in patients admitted to the oncology department of the Laquintinie Hospital in Douala.

Enteric infections of bacterial origin remain a cause of significant morbidity and mortality in cancer patients and are a common cause of public health problems in Cameroon [30–32]. In the present study, the maximum number of cancer patients in the age group above ≥60 years was 73.91%, followed by 69.66% and 67.14% in the age groups of [50–60[ years and [40–50[ years, respectively. This result is like that of Ngaroua et al., with an estimated prevalence of cancer in patients (51–60 years) of 54% in 2019 [33]. Because the mean age of patients in our study was considerably higher in cancer patients (49.54 ± 14.65 years) than in noncancer patients (41.53 ± 16.33 years), and the age went from 10 to 82 years by this study demonstrates that the incidence of cancer increases with age. It has been suggested that the poor survival of cancers in developing countries compared to some developed countries may be related to the advanced stage of diagnosis and the limited availability of adequate staging and treatment [34]. In our study, we discovered that 180 cancer patients exhibited tumor progression at stage 4, with a frequency of 58.63%, followed by stage 3 with 82 patients and a percentage of 26.71. This result could be due to multiple factors, such as poverty, a lack of screening programs, poor accessibility to diagnostic facilities, and cultural beliefs acting as barriers to early presentation [35].

The incidence of infections with antibiotic-resistant Enterobacteriaceae is increasing worldwide, particularly in cancer patients, posing a threat to public health and a challenge for doctors. The etiology of enteric disorders in cancer patients is multifactorial. We performed a bacteriological analysis of stool samples to look for the bacteria responsible for enteric infections. In the present study, bacteria were isolated from 150 (48.85%) cancer patients and 100 (50.00%) noncancer patients with enteric disorders. *Proteus mirabilis*, *Klebsiella oxytoca*, *Klebsiella pneumoniae*, *Salmonella typhi*, *Enterobacter cloacae*, and *Yersinia intemedia* were isolated more frequently in stool samples from cancer patients with values of 18 (56.43%), 37 (61.66%), 20 (66.66%), 13 (72.22%), 8 (80%), and 5 (100%), respectively, compared to noncancer patients with values of 14 (43.75%), 23 (38.34%), 10 (33.34%), 5 (27.28%), and 2 (20%), respectively, and *Yersinia intemedia* was not isolated from this group of patients. *Proteus* spp., *Klebsiella* spp., *Salmonella typhi*, and *Enterobacter cloacae* are among the bacteria responsible for various infections associated with cancer patients [36–38]. Antimicrobial susceptibility results showed that many of the bacteria isolated in this study were mainly susceptible to IMI, GEN, and AMK, while they were mainly resistant to ATM, cephalosporins (third or fourth generation), TET, and fluoroquinolone. Jiang et al. also demonstrated bacterial sensitivity in cancer patients [38]. *Klebsiella oxytoca* showed significantly higher rates of resistance in cancer patients than in noncancer patients to AMC (62.16% versus 30.43%), CTX (67.57% versus 34.78%), CAZ (75.68% versus 17.39%), COL (54.05% versus 34.78%), TET (83.78% versus 56.52%), and NIT (51.35% versus 13.04%). Ashour and El-Sharif studied antibiotic resistance in cancer patients, reporting a similar observation among cancer patients in Egypt [39].

This resistance is generally observed to be due to various mechanisms developed by Enterobacteriaceae, including enzymatic inactivation by the production of plasmid and chromosomal beta-lactamases, modification of the target, efflux, and violation of the membrane pathway [40–42]. The active release of antibiotics from the cell, or efflux, in Enterobacteriaceae is used to maintain resistance to tetracyclines, macrolides, and carbapenems [43]. The genes of the efflux system are located on plasmids and contribute to rapid propagation among Enterobacteriaceae [43]. Resistance to quinolones and fluoroquinolones is associated with the modification of topoisomerases II and IV, which are targets of these groups of antibiotics, reduced permeability across the bacterial outer membrane, and a reduction in the ability of the bacterial cells to produce efflux [43, 44].

Multidrug resistance in cancer patients is a major cause for concern. In the present study, the estimated rate of MDR isolates was remarkable (70.4%), among which the rate of MDR isolates in cancer patients was 80% compared to noncancer patients with a rate of MDR of 56%. *Klebsiella pneumonia* (85.00%), *Proteus mirabilis* (77.78%), *Salmonella typhi* (84.62%), *Klebsiella oxytoca* (86.49%), and *Proteus vulgaris* (84.78%) showed multidrug resistance in cancer patients. Resistance in *Salmonella typhi* isolates is a major problem in African countries in general and Cameroon in particular. Multidrug-resistant *Salmonella typhi* remains widespread in Africa, with resistance developing to an increasing number of antimicrobial classes and is becoming serious problem. MDR *S. typhi* and XDR *S. typhi* can originate from environmental samples, facilitating transmission to humans, particularly through the consumption of certain foods that are usually eaten raw [45]. The accumulation of mutations in the genes encoding the two targets of quinolones by DNA gyrase and topoisomerase IV, in *Salmonella typhi* is an important factor in resistance and reduced susceptibility to quinolones [46]. The MDR phenomenon may be due to the uncontrolled use of antibiotics in Cameroon. These results can be explained by an overexpression of efflux pumps, which play an important role in the MDR mechanism [40–42, 47]. This increase in multidrug efflux pumps is related to drug resistance because they may evacuate a wide spectrum of drugs, lowering antibiotic concentrations in the cell and promoting mutation accumulation [48].

Several recent studies have shown high levels of MDR pathogens in Cameroonian hospitals [49, 50]. In recent years, the emergence of MDR strains has been a growing problem in the country. The high rate of MDRs found in this study, particularly in cancer patients, may be explained by the fact that these patients had already been exposed to antibiotics. This high rate of MDR is also often correlated with numerous episodes of hospitalization and prolonged stays in the oncology unit of cancer patients. Chemotherapy can lead to a modification of the balance of the intestinal microbiota and a reduction in commensal anaerobic bacteria, thus contributing to an expansion of potentially pathogenic bacteria [51, 52]. The results of our study suggested a variation in the rate of multidrug resistance of bacterial isolates in cancer patients who have or have not undergone anticancer treatments. These results showed that *Proteus vulgaris* (56.41%), *Salmonella typhi* (90.90%), *Klebsiella oxytoca* (62.50%), and *Proteus mirabilis* (57.15%) had high multidrug resistance in cancer patients undergoing anticancer chemotherapeutic treatment. This could be due to the fact that anticancer chemotherapy promotes the emergence of antibiotic-resistant mutants from bacterial commensals in patients [13–19]. Many chemotherapeutic agents are likely to increase mutagenesis in bacteria and the emergence of de novo antimicrobial resistance through activation of the bacterial SOS response [20–24].

## 5. Conclusion

In the present study, it was shown that bacterial enteric infections in oncology patients were associated with a higher prevalence of multidrug resistance. Many of the bacteria isolated in this study were primarily susceptible to imipenem, gentamicin, and amikacin. The prevalence of MDR isolates was high, with a higher rate of MDR isolates in cancer patients than in noncancer patients. One likely explanation is selection pressure linked to treatment with large quantities of these antibiotics in cancer patients. The growing emergence of bacterial resistance to existing antibiotics requires the urgent development of new drugs. There is therefore an urgent need to limit antibiotic consumption in clinical practice, using narrow-spectrum antibiotics wherever possible on the basis of bacteriological stool culture reports. Finally, clinicians should focus more on enteric infections caused by MDR bacteria in cancer patients and take into account local epidemiological data on resistance when initiating antimicrobial therapy. Ongoing monitoring and surveillance are needed to better manage the problems caused by the rapid emergence of antimicrobial resistance. We question whether the method of antibiotic administration and the level of antimicrobial resistance in the community may have an impact on the emergence of resistance in intestinal Enterobacteriaceae during antibiotic treatment. Further studies comparing the effects of intravenous administration of cancer chemotherapy and antibiotics on intestinal flora are needed to evaluate this hypothesis. A limitation of this study is the lack of adjustment for risk factors associated with prior exposure to antibiotic therapy in the past year and the recollection of multidrug resistance. Furthermore, the data should not be generalized to the whole country. Despite these limitations, we remain convinced that this study provides essential information on the intestinal carriage of multidrug-resistant pathogenic bacteria in cancer patients.

## Figures and Tables

**Table 1:** Demographic characteristics of patients with enteric disorders included in the study.

Variable		Cancer patients *N* = 307 (%)	Noncancer patients *N* = 200 (%)	*p*-value

Sex	Male (*N* = 192)	109 (56.77)	83 (43.23)	0.102
	Female (*N* = 315)	198 (62.85)	117 (37.15)	

Age group per year	<20 years (*N* = 23)	11 (47.82)	12 (52.18)	0.001
	[20–30] years (*N* = 52)	16 (30.76)	36 (69.24)	
	[30–40] years (*N* = 88)	39 (44.31)	49 (55.69)	
	[40–50] years (*N* = 140)	94 (67.14)	46 (32.85)	
	[50–60] years (*N* = 89)	62 (69.66)	27 (30.34)	
	≥60 years (*N* = 115)	85 (73.91)	30 (26.09)	

Mean age; mean ± SD [min–max]	Total	49.54 ± 14.65 [10–82]	41.53 ± 16.33 [10–84]	0.001
	Male	50.01 ± 17.33 [10–82]	41.27 ± 15.61 [10–81]	
	Female	49.28 ± 12.99 [10–79]	41.70 ± 16.88 [10–84]	

*N*: number of patients.

**Table 2:** Distribution of different types of cancer according to cancer treatment.

Type of cancer	Cancer patients undergoing chemotherapy	Cancer patients without chemotherapy
Breast cancer *N* = 98	75 (39.89%)	23 (19.33%)
Cervical cancer *N* = 42	21 (11.17%)	21 (17.65%)
Stomach cancer *N* = 15	10 (5.32%)	5 (4.20%)
Cavum cancer *N* = 10	8 (4.26%)	2 (1.68%)
Cholangiocarcinoma *N* = 8	3 (1.60%)	5 (4.20%)
Colon/Rectal cancer *N* = 19	9 (4.79%)	10 (8.40%)
Liver cancer *N* = 14	0 (0.00%)	14 (11.76%)
Non-Hodgkin lymphoma *N* = 22	14 (7.45%)	8 (6.72%)
Lung cancer *N* = 8	7 (3.72%)	1 (0.84%)
Ovarian cancer *N* = 6	5 (2.66%)	1 (0.84%)
Pancreatic cancer *N* = 14	8 (4.26%)	6 (5.04%)
Prostate cancer *N* = 14	4 (2.13%)	10 (8.40%)
Kaposi sarcoma *N* = 14	11 (5.85%)	3 (2.52%)
Osteosarcoma *N* = 18	11 (5.85%)	7 (5.88%)
Esophageal cancer *N* = 5	2 (1.06%)	3 (2.52%)
Total *N* = 307	188 (61.24%)	119 (38.76%)

*N*: number of patients.

**Table 3:** Different risk factors for resistance in the study groups.

		Cancer patients	Noncancer patients	*X* ^2^ (*p*-value)
Previous exposure to antibiotics	Yes	203 (66.12%)	56 (28%)	70.34 (0.001)
	No	104 (33.88%)	144 (72%)	

Hospitalized patients	Yes	180 (58.63%)	0 (0.00%)	181.81 (0.001)
	No	127 (41.37%)	200 (100%)	

Chemotherapy	Less than four sessions	84 (44.58%)	ND	N/A
	More than five sessions	104 (55.32%)	ND	

ND: not defined N/A: not applicable *X*^2^: chi-square.

**Table 4:** Resistance profile of *Proteus mirabilis* and *Proteus vulgaris* depending on cancer status.

Antibiotics		*Proteus mirabilis n* = 32 (%)		*Proteus vulgaris n* = 92 (%)	
		Cancer + *n* = 18 (%)	Cancer − *n* = 14 (%)	Cancer + *n* = 46 (%)	Cancer − *n* = 46 (%)
IMP	S	18 (100)	13 (92.86)	46 (100)	45 (97.83)
	R	0 (0.00)	1 (7.14)	0 (0.00)	1 (2.17)

AMX	S	3 (16.67)	1 (7.14)	4 (8.70)	11 (23.91)
	R	15 (83.33)	13 (92.86)	42 (91.30)	35 (76.09)

AMC	S	5 (27.78)	9 (64.29)	5 (10.87)	27 (58.70)
	R	13 (72.22)	5 (35.71)	41 (89.13)	19 (41.30)

FOX	S	7 (38.39)	5 (35.71)	10 (21.74)	29 (63.04)
	R	11 (61.11)	9 (64.29)	36 (78.26)	17 (36.96)

CTX	S	10 (55.56)	6 (42.86)	8 (17.39)	29 (63.04)
	R	8 (44.44)	8 (57.14)	38 (82.61)	17 (36.96)

CXM	S	4 (22.22)	6 (42.86)	7 (15.22)	31 (67.39)
	I	0 (0.00)	0 (0.00)	0 (0.00)	1 (2.17)
	R	14 (77.80)	8 (57.14)	39 (84.78)	14 (30.43)

PRL	S	2 (11.11)	2 (14.29)	0 (0.00)	16 (34.78)
	R	16 (88.89)	12 (85.71)	46 (100)	30 (65.22)

CTR	S	8 (44.44)	8 (57.14)	17 (36.96)	28 (60.87)
	R	10 (55.56)	6 (42.86)	29 (63.04)	18 (39.13)

CAZ	S	5 (27.78)	9 (64.29)	7 (15.22)	33 (71.74)
	I	1 (5.56)	0 (0.00)	0 (0.00)	0 (0.00)
	R	12 (66.67)	5 (35.71)	39 (84.18)	13 (28.26)

AMK	S	18 (100)	11 (78.57)	40 (86.96)	40 (86.96)
	I	0 (0.00)	0 (0.00)	0 (0.00)	1 (2.17)
	R	0 (0.00)	3 (78.57)	6 (13.04)	5 (10.87)

GEN	S	17 (94.44)	13 (92.86)	41 (89.13)	45 (97.83)
	R	1 (5.56)	1 (7.14)	5 (10.87)	1 (2.17)

CIP	S	8 (44.44)	7 (50.00)	16 (34.78)	28 (60.87)
	I	0 (0.00)	1 (7.14)	0 (0.00)	4 (4.35)
	R	10 (55.56)	6 (42.86)	36 (65.22)	16 (34.18)

OFX	S	7 (38.89)	8 (57.14)	19 (41.30)	26 (56.52)
	I	0 (0.00)	0 (0.00)	0 (0.00)	4 (8.70)
	R	11 (61.11)	6 (42.86)	27 (58.70)	16 (34.78)

NAL	S	6 (33.33)	9 (64.29)	12 (26.09)	20 (43.48)
	R	12 (66.67)	5 (35.71)	34 (73.91)	26 (56.52)

COT	S	5 (27.78)	10 (71.43)	27 (58.70)	30 (65.22)
	I	0 (0.00)	0 (0.00)	1 (2.17)	1 (2.17)
	R	13 (72.22)	4 (28.57)	18 (39.13)	15 (32.61)

COL	S	5 (27.78)	7 (50.00)	14 (30.43)	32 (69.57)
	R	13 (72.22)	7 (50.00)	32 (69.57)	14 (30.43)

TET	S	2 (11.11)	6 (42.86)	3 (6.52)	17 (36.96)
	R	16 (88.89)	8 (57.14)	43 (93.48)	29 (63.04)

VAN	S	1 (5.56)	2 (14.29)	1 (2.17)	22 (41.83)
	R	17 (94.44)	12 (85.71)	45 (97.83)	24 (52.17)

NIT	S	7 (38.89)	10 (71.48)	17 (36.96)	37 (80.43)
	R	11 (61.11)	4 (28.57)	29 (63.04)	9 (19.57)

ATM	S	10 (55.56)	9 (64.29)	21 (45.65)	33 (71.74)
	I	0 (0.00)	1 (7.14)	0 (0.00)	2 (4.35)
	R	8 (44.44)	4 (28.57)	25 (54.35)	11 (23.91)

FOS	S	9 (50.00)	9 (64.29)	28 (60.87)	41 (89.13)
	R	9 (50.00)	5 (35.71)	18 (39.13)	5 (10.87)

ERY	S	7 (38.89)	5 (35.71)	5 (10.87)	31 (67.39)
	I	0 (0.00)	0 (0.00)	0 (0.00)	1 (2.17)
	R	11 (61.11)	9 (64.29)	41 (89.13)	14 (30.43)

IPM: imipenem, AMX: amoxicillin, AMC: amoxiclav, CAZ: ceftazidim, FOX: cefoxitin, CTX: cefotaxim, CXM: cefuroxim, PRL: piperacillin, CTR: ceftriazone AMK: amikacin, GEN: gentamicin, CIP: ciprofloxacin, OFX: ofloxacin, NAL: naxidilic acid, COT: trimethoprim-sulfamethoxazole, COL: colistin, TET: tetracyclin, VAN: vancomycin NIT: nitrofurantoin, ATM: aztreonam, FOS: fosfomycin, ERY: erythromycin, S: sensitive, I: intermediate, R: resistant.

**Table 5:** Resistance profile of other bacteria depending on cancer status.

Antibiotics		*Klebsiella oxytoca n* = 60 (%)		*Klebsiella pneumoniae n* = 30 (%)		*Salmonella typhi n* = 18 (%)		*Enterobacter cloasae n* = 10 (%)		*Yersinia intemedia n* = 05 (%)	*Serratia odorifera n* = 03 (%)
		Cancer + *n* = 37 (%)	Cancer − *n* = 23 (%)	Cancer + *n* = 20 (%)	Cancer − *n* = 10 (%)	Cancer + *n* = 13 (%)	Cancer − *n* = 05 (%)	Cancer + *n* = 08 (%)	Cancer − *n* = 02 (%)	Cancer + *n* = 05 (%)	Cancer + *n* = 03 (%)
IMP	S	35 (94.59)	23 (100)	20 (100)	9 (90.00)	12 (92.31)	5 (100)	8 (100)	2 (100)	5 (100)	3 (100)
	R	2 (5.41)	0 (0.00)	0 (0.00)	1 (10.00)	1 (7.69)	0 (0.00)	0 (0.00)	0 (0.00)	0 (0.00)	0 (0.00)

AMX	S	5 (13.51)	5 (21.74)	1 (5.00)	1 (10.00)	0 (0.00)	0 (0.00)	1 (12.50)	1 (50.00)	0 (0.00)	1 (33.33)
	I	0 (0.00)	0 (0.00)	1 (5.00)	0 (0.00)	0 (0.00)	0 (0.00)	0 (0.00)	0 (0.00)	0 (0.00)	0 (0.00)
	R	32 (86.49)	18 (78.26)	18 (90.0)	9 (90.00)	13 (100)	5 (100)	7 (87.50)	1 (50.00)	5 (100)	2 (66.67)

AMC	S	14 (37.84)	16 (69.57)	8 (40.00)	6 (60.00)	4 (30.77)	2 (40.00)	1 (12.50)	1 (50.00)	0 (0.00)	1 (3.33)
	R	23 (62.16)	7 (30.43)	12 (60.0)	4 (40.00)	9 (69.23)	3 (60.00)	7 (87.50)	1 (50.00)	5 (100)	2 (66.67)

FOX	S	10 (27.03)	14 (60.87)	4 (20.0)	6 (60.00)	2 (15.38)	4 (80.00)	4 (50.00)	2 (100)	2 (40.00)	0 (0.00)
	R	27 (72.97)	9 (39.13)	16 (80.0)	4 (40.00)	11 (84.62)	1 (20.00)	4 (50.00)	0 (0.00)	3 (60.00)	3 (100)

CTX	S	12 (32.43)	15 (65.22)	3 (15.00)	5 (50.00)	4 (30.77)	2 (40.00)	4 (50.00)	2 (100)	1 (20.00)	1 (33.33)
	R	25 (67.57)	8 (34.78)	17 (85.0)	5 (50.00)	9 (69.23)	3 (60.00)	4 (50.00)	0 (0.00)	4 (80.00)	2 (66.67)

CXM	S	8 (21.62)	15 (65.22)	2 (10.00)	5 (50.00)	0 (0.00)	2 (40.00)	3 (37.50)	2 (100)	0 (0.00)	1 (33.33)
	R	29 (78.38)	8 (34.78)	18 (90.0)	5 (50.00)	13 (100)	3 (60.00)	5 (62.50)	0 (0.00)	5 (100)	2 (66.67)

PRL	S	2 (5.41)	11 (74.83)	0 (0.00)	1 (10.00)	0 (0.00)	0 (0.00)	0 (0.00)	0 (0.00)	0 (0.00)	0 (0.00)
	R	35 (94.59)	12 (52.17)	20 (100)	9 (90.00)	13 (100)	5 (100)	8 (100)	2 (100)	5 (100)	3 (100)

CTR	S	14 (37.84)	14 (60.87)	5 (25.00)	7 (70.00)	6 (46.15)	2 (40.00)	2 (25.00)	1 (50.00)	2 (40.00)	0 (0.00)
	R	23 (62.16)	9 (39.13)	15 (75.0)	3 (30.00)	7 (53.85)	3 (60.00)	6 (75.00)	1 (50.00)	3 (60.00)	3 (100)

CAZ	S	9 (24.32)	19 (82.61)	3 (15.00)	6 (60.00)	2 (15.38)	4 (80.00)	4 (50.00)	2 (100)	1 (20.00)	1 (3.33)
	I	0 (0.00)	0 (0.00)	2 (10.00)	0 (0.00)	0 (0.00)	0 (0.00)	0 (0.00)	0 (0.00)	0 (0.00)	0 (0.00)
	R	28 (75.68)	4 (17.39)	15 (85.0)	4 (40.00)	11 (84.62)	1 (20.00)	4 (50.00)	0 (0.00)	4 (80.00)	2 (66.67)

AMK	S	34 (91.89)	22 (95.65)	20 (100)	9 (90.00)	11 (84.62)	5 (100)	8 (100)	2 (100)	5 (100)	3 (100)
	R	3 (8.11)	1 (4.35)	0 (0.00)	1 (10.00)	2 (15.38)	0 (0.00)	0 (0.00)	0 (0.00)	0 (0.00)	0 (0.00)

GEN	S	37 (100)	21 (91.30)	20 (100)	10 (100)	13 (100)	5 (100)	8 (100)	2 (100)	3 (60.00)	3 (100)
	R	0 (0.00)	2 (8.70)	0 (0.00)	0 (0.00)	0 (0.00)	0 (0.00)	0 (0.00)	0 (0.00)	2 (40.00)	0 (0.00)

CIP	S	15 (40.54)	13 (52.52)	10 (50.0)	5 (50.00)	6 (46.15)	2 (40.00)	3 (37.50)	1 (50.00)	2 (40.00)	1 (33.33)
	I	0 (0.00)	1 (4.35)	1 (5.00)	1 (10.00)	0 (0.00)	0 (0.00)	0 (0.00)	0 (0.00)	0 (0.00)	0 (0.00)
	R	22 (59.46)	9 (39.13)	9 (45.00)	4 (40.00)	7 (53.85)	3 (60.00)	5 (62.50)	1 (50.00)	3 (60.00)	2 (66.67)

OFX	S	15 (40.54)	13 (52.52)	10 (50.0)	5 (50.00)	6 (46.15)	2 (40.00)	3 (37.50)	1 (50.00)	2 (40.00)	1 (33.33)
	I	0 (0.00)	1 (4.35)	0 (0.00)	1 (10.00)	0 (0.00)	0 (0.00)	0 (0.00)	0 (0.00)	0 (0.00)	0 (0.00)
	R	22 (59.46)	9 (39.13)	10 (50.0)	4 (40.00)	7 (53.85)	3 (60.00)	5 (62.50)	1 (50.00)	3 (60.00)	2 (66.67)

NAL	S	13 (35.14)	10 (43.48)	10 (50.0)	2 (20.00)	2 (13.38)	0 (0.00)	1 (12.50)	1 (50.00)	0 (0.00)	1 (33.33)
	R	24 (64.86)	13 (56.52)	10 (50.0)	8 (80.00)	11 (84.62)	5 (100)	7 (87.50)	1 (50.00)	5 (100)	2 (66.67)

COT	S	24 (64.86)	15 (65.22)	13 (65.0)	7 (70.00)	6 (46.15)	0 (0.00)	5 (62.50)	2 (100)	2 (40.00)	1 (33.33)
	R	13 (35.14)	8 (34.78)	7 (35.00)	3 (30.00)	7 (53.85)	5 (100)	3 (37.50)	0 (0.00)	3 (60.00)	2 (66.67)

COL	S	17 (45.95)	15 (65.22)	8 (40.00)	9 (90.00)	4 (30.77)	5 (100)	1 (12.50)	1 (50.00)	0 (0.00)	3 (100)
	R	20 (54.05)	8 (34.78)	12 (60.0)	1 (10.00)	9 (69.23)	0 (0.00)	7 (87.50)	1 (50.00)	5 (100)	0 (0.00)

TET	S	6 (16.22)	10 (43.48)	3 (15.00)	6 (60.00)	2 (15.38)	0 (0.00)	1 (12.50)	2 (100)	0 (0.00)	1 (33.33)
	R	31 (83.78)	13 (56.52)	17 (85.0)	4 (40.00)	11 (84.62)	5 (100)	7 (87.50)	0 (0.00)	5 (100)	2 (66.67)

VAN	S	3 (8.11)	15 (65.22)	0 (0.00)	7 (70.00)	0 (0.00)	2 (40.00)	0 (0.00)	0 (0.00)	0 (0.00)	1 (33.33)
	R	34 (91.89)	8 (34.78)	20 (100)	3 (30.00)	13 (100)	3 (60.00)	8 (100)	2 (100)	5 (100)	2 (66.67)

NIT	S	18 (48.65)	20 (86.96)	12 (60.0)	7 (70.00)	3 (23.08)	5 (100)	6 (75.00)	2 (100)	1 (20.00)	1 (33.33)
	R	19 (51.35)	3 (13.04)	8 (40.00)	3 (30.00)	10 (76.92)	0 (0.00)	2 (25.00)	0 (0.00)	4 (80.00)	2 (66.67)

ATM	S	17 (45.95)	16 (69.57)	13 (65.0)	7 (70.00)	6 (46.15)	4 (80.00)	4 (50.00)	2 (100)	0 (0.00)	1 (33.33)
	I	1 (2.70)	0 (0.00)	0 (0.00)	0 (0.00)	0 (0.00)	0 (0.00)	0 (0.00)	0 (0.00)	0 (0.00)	0 (0.00)
	R	19 (51.35)	7 (30.43)	7 (35.00)	3 (30.00)	7 (53.85)	1 (20.00)	4 (50.00)	0 (0.00)	5 (100)	2 (66.67)

FOS	S	21 (56.76)	16 (72.73)	14 (70.0)	9 (90.00)	6 (46.15)	4 (80.00)	4 (50.00)	2 (100)	3 (60.00)	1 (33.33)
	I	0 (0.00)	1 (4.55)	0 (0.00)	0 (0.00)	0 (0.00)	0 (0.00)	0 (0.00)	0 (0.00)	0 (0.00)	0 (0.00)
	R	16 (43.24)	5 (22.73)	6 (30.00)	1 (10.00)	7 (53.85)	1 (20.00)	4 (50.00)	0 (0.00)	2 (40.00)	2 (66.67)

ERY	S	6 (16.22)	19 (82.61)	4 (20.00)	4 (40.00)	1 (7.69)	2 (40.00)	2 (25.00)	2 (100)	0 (0.00)	1 (33.33)
	I	0 (0.00)	0 (0.00)	16 (80.0)	0 (0.00)	0 (0.00)	0 (0.00)	0 (0.00)	0 (0.00)	0 (0.00)	0 (0.00)

IPM: imipenem, AMX: amoxicillin, AMC: amoxiclav, CAZ: ceftazidim, FOX: cefoxitin, CTX: cefotaxim, CXM: cefuroxim, PRL: piperacillin, CTR: ceftriazone AMK: amikacin, GEN: gentamicin, S: sensitive, I: intermediate, R: resistant, CIP: ciprofloxacin, OFX: ofloxacin, NAL: nalidixic acid, COT: trimethoprim-sulfamethoxazole, COL: colistin, TET: tetracycline, VAN: vancomycin NIT: nitrofurantoin, ATM: aztreonam, FOS: fosfomycin, ERY: erythromycin, S: sensitive, I: intermediate, R: resistant.

**Table 6:** Association of prior exposure to antibiotic therapy with multidrug resistance of *Proteus vulgaris* following bivariate logistic regression analysis.

		MDR *P. vulgaris n* = 66 (%)	Non-MDR *P. vulgaris n* = 26 (%)	OR (95% CI)	*p*-value
Cancer patients	Previous exposure to antibiotic therapy	28 (42.22)	3 (11.53)	2.33 (0.410–13.258)	0.296
	No previous exposure to antibiotic therapy	12 (18.18)	3 (11.53)		

Noncancer patients	Previous exposure to antibiotic therapy	8 (12.12)	5 (19.23)	1.33 (0.359–4.945)	0.462
	No previous exposure to antibiotic therapy	18 (27.27)	12 (46.15)		

OR: odds ratio CI: confidence interval MDR: multidrug resistance *P. vulgaris: Proteus vulgaris*.

**Table 7:** Association of prior exposure to antibiotic therapy with multidrug resistance of *Klebsiella oxytoca* following bivariate logistic regression analysis.

		MDR *K. oxytoca n* = 42 (%)	Non-MDR *K. oxytoca n* = 18 (%)	OR (95% CI)	*p*-value
Cancer patients	Previous exposure to antibiotic therapy	23 (54.22)	3 (16.66)	1.70 (0.242–11.952)	0.471
	No previous exposure to antibiotic therapy	9 (21.42)	2 (11.11)		

Noncancer patients	Previous exposure to antibiotic therapy	9 (21.42)	8 (44.44)	1.11 (0.161–7.632)	0.638
	No previous exposure to antibiotic therapy	1 (2.38)	5 (27.77)		

OR: odds ratio CI: confidence interval MDR: multidrug resistance *K. oxytoca: Klebsiella oxytoca*.

**Table 8:** Association of prior exposure to antibiotic therapy with multidrug resistance of *Klebsiella pneumoniae* following bivariate logistic regression analysis.

		MDR *K. pneumoniae n* = 19 (%)	Non-MDR *K. pneumoniae n* = 11 (%)	OR (95% CI)	*p*-value
Cancer patients	Previous exposure to antibiotic therapy	13 (68.42)	2 (18.18)	1.62 (0.114–22.982)	0.600
	No previous exposure to antibiotic therapy	1 (5.26)	4 (36.36)		

Noncancer patients	Previous exposure to antibiotic therapy	1 (5.26)	2 (18.18)	1.50 (0.088–25.395)	0.666
	No previous exposure to antibiotic therapy	4 (21.05)	3 (27.77)		

OR: odds ratio CI: confidence interval MDR: multidrug resistance *K. pneumoniae: Klebsiella pneumoniae*.

## Data Availability

All data generated or analyzed during this study are included in this published article and the supporting file.

## Supplementary Materials

Supplementary Materials Supplementary file (.PDF): All raw data generated to support the results of this study. S1: Participants and their Features; S2: Patients and cancer treatments; S3: Patients with *K. pneumoniae* infection and antibiogram; S4: Patients with *K. oxytoca* infection and antibiogram; S5: Patients with P. mirabilis infection and antibiogram; S6: Patients with *P. vulgaris* infection and antibiogram; S7: Patients with *E. cloacae* infection and antibiogram; S8: Patients with *Y. intermedia* infection and antibiogram; S9: Patients with *S. typhi* infection and antibiogram; S10: Patients with *S. odorifera* infection and antibiogram.
