# Supplementary material for: Multidrug Resistant Enteric Bacteria from Cancer Patients Admitted in Douala Laquintinie Hospital, Littoral Region of Cameroon
Source: Can J Infect Dis Med Microbiol. 2024 Jul 12;2024:2084884. doi: 10.1155/2024/2084884 (PMC11259499; doi:10.1155/2024/2084884)
Supplement: Supplementary Materials — Supplementary file (.PDF): All raw data generated to support the results of this study. S1: Participants and their Features; S2: Patients and cancer treatments; S3: Patients with K. pneumoniae infection and antibiogram; S4: Patients with K. oxytoca infection and antibiogram; S5: Patients with P. mirabilis infection and antibiogram; S6: Patients with P. vulgaris infection and antibiogram; S7: Patients with E. cloacae infection and antibiogram; S8: Patients with Y. intermedia infection and antibiogram; S9: Patients with S. typhi infection and antibiogram; S10: Patients with S. odorifera infection and antibiogram. [file 2084884.f1.pdf]

Multidrug resistant enteric bacteria from cancer patients admitted in Douala Laquintinie Hospital, Littoral Region of Cameroon

Michael Francis Kengne[a], *Ornella D. Tsobeng*[a], *Ballue S. T. Dadjo*[a], *Victor Kuete*[a*], *Armelle T. Mbaveng*[a]**,

[a]*Department of Biochemistry, Faculty of Science, University of Dschang, Dschang, Cameroon*

Corresponding author:

**Email: kuetevictor@yahoo.fr; ORCID: http://orcid.org/0000-0002-1070-1236 (Victor Kuete)*

***Email: armbatsa@yahoo.fr; ORCID: https://orcid.org/0000-0003-4178-4967 (Armelle T. Mbaveng)*

Other authors emails:

Email: fmkengne@yahoo.com (Michael Francis Kengne)

Email: tsobengornella98@gmail.com (Ornella D. Tsobeng)

Email: ballueserges@gmail.com (Ballue S. T. Dadjo)

S1. Participants and their Features

Patient code	Patient features					
	SEX	AGE	Age range	Education level	Occupation	Marital status
K001	M	30	[30-40[	Secondary school	Private sector	Bachelor
K002	M	54	[50-60[	Primary school	Private sector	Bride
K003	F	50	[50-60[	Secondary school	Household	Bride
K004	F	49	[40-50[	Primary school	Household	Bride
K005	M	31	[30-40[	Higher education	Private sector	Bachelor
K006	M	59	[50-60[	Secondary school	Private sector	Bride
K007	F	67	≥60	Primary school	Household	Widow(er)
K008	M	38	[30-40[	Secondary school	Jobless	Bachelor
K009	F	42	[40-50[	Secondary school	Private sector	Widow(er)
K010	F	43	[40-50[	Secondary school	Private sector	Bride
K011	F	41	[40-50[	Secondary school	Household	Bride
K012	M	82	≥60	Analphabet	Private sector	Bride
K013	F	54	[50-60[	Primary school	Household	Widow(er)
K014	M	18	<20	Secondary school	Student	Bachelor
K015	F	33	[30-40[	Higher education	Private sector	Bride
K016	M	42	[40-50[	Higher education	Private sector	Bride
K017	M	68	≥60	Primary school	Private sector	Bride
K018	M	66	≥60	Analphabet	Jobless	Bride
K019	M	70	≥60	Analphabet	Private sector	Bride
K020	F	43	[40-50[	Secondary school	Household	Bride
K021	M	61	≥60	Secondary school	Jobless	Bride
K022	M	64	≥60	Higher education	Jobless	Bride
K023	M	57	[50-60[	Secondary school	Jobless	Bride

K024	M	53	[50-60[	Secondary school	Private sector	Bachelor
K025	F	64	≥60	Primary school	Household	Widow(er)
K026	M	29	[20-30[	Secondary school	Private sector	Bachelor
K027	M	75	≥60	Primary school	Jobless	Bride
K028	F	62	≥60	Primary school	Household	Bride
K029	F	58	[50-60[	Secondary school	Household	Bride
K030	F	40	[40-50[	Secondary school	Household	Bachelor
K031	M	65	≥60	Secondary school	Jobless	Bride
K032	F	50	[50-60[	Secondary school	Household	Bride
K033	F	27	[20-30[	Secondary school	Private sector	Bachelor
K034	F	38	[30-40[	Higher education	Private sector	Bachelor
K035	F	43	[40-50[	Secondary school	Household	Bride
K036	F	52	[50-60[	Higher education	Household	Bride
K037	F	40	[40-50[	Secondary school	Household	Bride
K038	M	43	[40-50[	Primary school	Jobless	Bride
K039	M	74	≥60	Analphabet	Jobless	Bride
K040	M	75	≥60	Primary school	Jobless	Bride
K041	M	51	[50-60[	Secondary school	Private sector	Widow(er)
K042	F	74	≥60	Analphabet	Household	Widow(er)
K043	F	72	≥60	Analphabet	Household	Widow(er)
K044	F	43	[40-50[	Secondary school	Private sector	Bride
K045	M	56	[50-60[	Secondary school	Private sector	Bride
K046	F	67	≥60	Analphabet	Private sector	Widow(er)
K047	M	43	[40-50[	Secondary school	Private sector	Bride
K048	F	55	[50-60[	Secondary school	Household	Widow(er)
K049	F	50	[50-60[	Secondary school	Household	Bride
K050	M	50	[50-60[	Higher education	Private sector	Bride
K051	F	42	[40-50[	Secondary school	Private sector	Bachelor
K052	F	45	[40-50[	Secondary school	Household	Bride

K053	F	47	[40-50[	Secondary school	Private sector	Bride
K054	F	63	≥60	Secondary school	Household	Widow(er)
K055	F	10	<20	Primary school	Student	Bachelor
K056	F	69	≥60	Primary school	Private sector	Bride
K057	M	66	≥60	Secondary school	Private sector	Bride
K058	F	59	[50-60[	Analphabet	Private sector	Widow(er)
K059	M	60	≥60	Secondary school	Private sector	Bride
K060	F	56	[50-60[	Secondary school	Household	Bride
K061	F	40	[40-50[	Secondary school	Household	Bride
K062	F	38	[30-40[	Primary school	Household	Bride
K063	M	78	≥60	Secondary school	Jobless	Bride
K064	F	60	≥60	Primary school	Private sector	Bride
K065	M	68	≥60	Secondary school	Private sector	Bride
K066	M	77	≥60	Analphabet	Private sector	Bride
K067	M	39	[30-40[	Higher education	Private sector	Bride
K068	F	36	[30-40[	Secondary school	Private sector	Bachelor
K069	M	34	[30-40[	Secondary school	Jobless	Bachelor
K070	M	29	[20-30[	Secondary school	Private sector	Bachelor
K071	M	55	[50-60[	Secondary school	Private sector	Divorced
K072	F	57	[50-60[	Secondary school	Private sector	Divorced
K073	F	41	[40-50[	Secondary school	Household	Bride
K074	F	23	[20-30[	Secondary school	Student	Bachelor
K075	F	65	≥60	Secondary school	Household	Widow(er)
K076	F	75	≥60	Primary school	Household	Widow(er)
K077	M	47	[40-50[	Primary school	Private sector	Bride
K078	M	57	[50-60[	Primary school	Private sector	Bride
K079	F	50	[50-60[	Higher education	Private sector	Bride
K080	F	41	[40-50[	Secondary school	Jobless	Bachelor
K081	F	60	≥60	Secondary school	Household	Bride

K082	F	51	[50-60[	Secondary school	Household	Bride
K083	F	49	[40-50[	Secondary school	Household	Bride
K084	M	70	≥60	Higher education	Private sector	Bride
K085	F	44	[40-50[	Secondary school	Household	Bride
K086	M	70	≥60	Secondary school	Jobless	Bride
K087	F	67	≥60	Secondary school	Household	Bride
K088	F	44	[40-50[	Secondary school	Household	Bride
K089	M	75	≥60	Secondary school	Jobless	Bride
K090	M	50	[50-60[	Secondary school	Private sector	Bride
K091	M	58	[50-60[	Analphabet	Household	Bride
K092	F	37	[30-40[	Secondary school	Household	Bride
K093	F	48	[40-50[	Secondary school	Private sector	Bride
K094	M	33	[30-40[	Secondary school	Private sector	Bachelor
K095	F	50	[50-60[	Secondary school	Private sector	Bride
K096	M	69	≥60	Secondary school	Jobless	Bride
K097	F	48	[40-50[	Secondary school	Household	Bride
K098	F	15	<20	Secondary school	Student	Bachelor
K099	M	58	[50-60[	Secondary school	Jobless	Bride
K100	F	52	[50-60[	Secondary school	Household	Bride
K101	F	29	[20-30[	Secondary school	Private sector	Bachelor
K102	M	13	<20	Primary school	Student	Bachelor
K103	M	32	[30-40[	Secondary school	Private sector	Bachelor
K104	F	54	[50-60[	Secondary school	Household	Bachelor
K105	F	56	[50-60[	Secondary school	Private sector	Widow(er)
K106	F	41	[40-50[	Secondary school	Household	Bride
K107	M	11	<20	Primary school	Student	Bachelor
K108	F	44	[40-50[	Secondary school	Private sector	Bride
K109	F	43	[40-50[	Secondary school	Household	Bride
K110	F	38	[30-40[	Secondary school	Household	Bride

K111	F	12	<20	Primary school	Student	Bachelor
K112	F	41	[40-50[	Secondary school	Household	Bride
K113	M	57	[50-60[	Secondary school	Jobless	Bride
K114	F	38	[30-40[	Secondary school	Private sector	Bride
K115	F	54	[50-60[	Secondary school	Household	Bride
K116	F	44	[40-50[	Secondary school	Private sector	Bride
K117	F	50	[50-60[	Secondary school	Household	Bride
K118	M	71	≥60	Secondary school	Jobless	Bride
K119	F	68	≥60	Analphabet	Household	Bride
K120	F	61	≥60	Analphabet	Household	Widow(er)
K121	F	60	≥60	Analphabet	Household	Widow(er)
K122	F	49	[40-50[	Secondary school	Private sector	Bride
K123	F	39	[30-40[	Secondary school	Household	Bride
K124	M	37	[30-40[	Secondary school	Private sector	Bride
K125	F	64	≥60	Secondary school	Household	Bride
K126	F	36	[30-40[	Secondary school	Household	Bachelor
K127	F	45	[40-50[	Secondary school	Private sector	Bride
K128	F	50	[50-60[	Secondary school	Household	Bride
K129	F	30	[30-40[	Secondary school	Household	Bride
K130	F	45	[40-50[	Secondary school	Private sector	Bachelor
K131	F	47	[40-50[	Primary school	Household	Bride
K132	F	76	≥60	Analphabet	Household	Widow(er)
K133	F	40	[40-50[	Higher education	Private sector	Bride
K134	F	27	[20-30[	Higher education	Private sector	Bride
K135	M	80	≥60	Analphabet	Jobless	Bride
K136	F	55	[50-60[	Analphabet	Household	Bride
K137	M	11	<20	Primary school	Student	Bachelor
K138	F	50	[50-60[	Analphabet	Household	Bride
K139	M	10	<20	Primary school	Student	Bachelor

K140	M	63	≥60	Primary school	Private sector	Bride
K141	F	19	<20	Secondary school	Student	Bachelor
K142	M	42	[40-50[	Secondary school	Private sector	Bride
K143	M	32	[30-40[	no scolarise	Jobless	Bride
K144	M	46	[40-50[	Secondary school	Private sector	Bride
K145	F	54	[50-60[	Primary school	Private sector	Bride
K146	F	43	[40-50[	Secondary school	Household	Bachelor
K147	M	58	[50-60[	Primary school	Private sector	Bride
K148	F	72	≥60	Analphabet	Household	Widow(er)
K149	F	49	[40-50[	Secondary school	Household	Bride
K150	F	79	≥60	Analphabet	Household	Widow(er)
K151	F	62	≥60	Primary school	Private sector	Bride
K152	F	66	≥60	Analphabet	Household	Widow(er)
K153	F	66	≥60	Analphabet	Household	Bride
K154	F	51	[50-60[	Primary school	Household	Bachelor
K155	F	46	[40-50[	Secondary school	Private sector	Bachelor
K156	F	60	≥60	Primary school	Household	Bride
K157	F	68	≥60	Secondary school	Household	Bride
K158	M	61	≥60	Secondary school	Private sector	Bride
K159	M	45	[40-50[	Secondary school	Jobless	Bachelor
K160	F	47	[40-50[	Secondary school	Household	Bachelor
K161	F	32	[30-40[	Higher education	Private sector	Bachelor
K162	F	54	[50-60[	Analphabet	Private sector	Bride
K163	F	64	≥60	Analphabet	Household	Bachelor
K164	F	48	[40-50[	Secondary school	Household	Bride
K165	M	43	[40-50[	Secondary school	Private sector	Bride
K166	M	64	≥60	Secondary school	Jobless	Bride
K167	F	60	≥60	Primary school	Household	Bride
K168	M	56	[50-60[	Primary school	Private sector	Bride

K169	M	47	[40-50[	Secondary school	Private sector	Bride
K170	F	56	[50-60[	Secondary school	Household	Bride
K171	F	39	[30-40[	Higher education	Private sector	Bride
K172	F	31	[30-40[	Secondary school	Household	Bachelor
K173	M	57	[50-60[	Secondary school	Private sector	Bride
K174	M	54	[50-60[	Secondary school	Jobless	Bride
K175	M	28	[20-30[	Secondary school	Student	Bachelor
K176	M	45	[40-50[	Primary school	Private sector	Bride
K177	M	62	≥60	Primary school	Private sector	Bride
K178	M	62	≥60	Secondary school	Private sector	Bride
K179	M	55	[50-60[	Secondary school	Private sector	Bride
K180	F	46	[40-50[	Secondary school	Private sector	Bachelor
K181	M	42	[40-50[	Primary school	Private sector	Bride
K182	M	72	≥60	Primary school	Jobless	Bride
K183	F	45	[40-50[	Secondary school	Private sector	Bride
K184	M	69	≥60	Secondary school	Jobless	Bride
K185	F	36	[30-40[	Higher education	Private sector	Bride
K186	M	41	[40-50[	Secondary school	Household	Bride
K187	F	47	[40-50[	Secondary school	Household	Bride
K188	M	67	≥60	Analphabet	Private sector	Bride
K189	F	63	≥60	Analphabet	Household	Bride
K190	F	66	≥60	Secondary school	Household	Bachelor
K191	M	28	[20-30[	Secondary school	Private sector	Bachelor
K192	M	54	[50-60[	Secondary school	Private sector	Bride
K193	M	41	[40-50[	Secondary school	Private sector	Bride
K194	F	36	[30-40[	Primary school	Private sector	Bachelor
K195	M	48	[40-50[	Higher education	Private sector	Bride
K196	F	64	≥60	Analphabet	Household	Bachelor
K197	M	60	≥60	Secondary school	Private sector	Bride

K198	M	45	[40-50[	Secondary school	Private sector	Bachelor
K199	M	41	[40-50[	Primary school	Private sector	Bachelor
K200	F	54	[50-60[	Secondary school	Household	Bride
K201	M	37	[30-40[	Secondary school	Private sector	Bachelor
K202	M	39	[30-40[	Secondary school	Private sector	Bachelor
K203	M	43	[40-50[	Primary school	Jobless	Bachelor
K204	F	60	≥60	Primary school	Household	Bachelor
K205	F	43	[40-50[	Secondary school	Private sector	Bride
K206	F	40	[40-50[	Primary school	Household	Bride
K207	M	44	[40-50[	Analphabet	Private sector	Bride
K208	F	61	≥60	Secondary school	Household	Bride
K209	F	56	[50-60[	Primary school	Household	Bride
K210	F	31	[30-40[	Secondary school	Household	Bachelor
K211	F	62	≥60	Secondary school	Household	Bride
K212	M	52	[50-60[	Higher education	Private sector	Bride
K213	F	63	≥60	Secondary school	Household	Bride
K214	F	39	[30-40[	Secondary school	Private sector	Bride
K215	F	45	[40-50[	Higher education	Household	Bride
K216	M	44	[40-50[	Higher education	Private sector	Bride
K217	F	31	[30-40[	Higher education	Private sector	Bride
K218	F	47	[40-50[	Higher education	Private sector	Bride
K219	F	36	[30-40[	Secondary school	Private sector	Bachelor
K220	F	28	[20-30[	Secondary school	Private sector	Bachelor
K221	F	47	[40-50[	Secondary school	Household	Bride
K222	F	42	[40-50[	Secondary school	Private sector	Bride
K223	F	72	≥60	Primary school	Household	Widow(er)
K224	F	49	[40-50[	Secondary school	Household	Bride
K225	F	36	[30-40[	Secondary school	Private sector	Bride

K226	F	45	[40-50[	Secondary school	Household	Bride
K227	F	75	≥60	Analphabet	Household	Widow(er)
K228	F	29	[20-30[	Secondary school	Household	Bride
K229	M	46	[40-50[	Primary school	Private sector	Bride
K230	F	45	[40-50[	Secondary school	Private sector	Bride
K231	F	54	[50-60[	Secondary school	Private sector	Bride
K232	F	47	[40-50[	Secondary school	Private sector	Bride
K233	F	49	[40-50[	Analphabet	Household	Bride
K234	F	36	[30-40[	Secondary school	Private sector	Bachelor
K235	F	40	[40-50[	Secondary school	Private sector	Bachelor
K236	M	81	≥60	Analphabet	Jobless	Bride
K237	F	61	≥60	Analphabet	Private sector	Widow(er)
K238	F	68	≥60	Analphabet	Household	Widow(er)
K239	F	20	[20-30[	Secondary school	Student	Bachelor
K240	M	80	≥60	Analphabet	Jobless	Bride
K241	F	56	[50-60[	Secondary school	Private sector	Bride
K242	F	39	[30-40[	Secondary school	Private sector	Bride
K243	F	45	[40-50[	Higher education	Private sector	Bachelor
K244	F	38	[30-40[	Secondary school	Private sector	Bachelor
K245	F	33	[30-40[	Primary school	Household	Bride
K246	F	76	≥60	Secondary school	Jobless	Widow(er)
K247	F	62	≥60	Analphabet	Household	Bride
K248	F	44	[40-50[	Secondary school	Private sector	Bachelor
K249	F	43	[40-50[	Secondary school	Private sector	Bride
K250	F	50	[50-60[	Secondary school	Household	Bride
K251	F	45	[40-50[	Secondary school	Household	Bachelor
K252	M	14	<20	Secondary school	Student	Bachelor
K253	F	43	[40-50[	Secondary school	Household	Bachelor
K254	F	61	≥60	Secondary school	Household	Bride

K255	M	71	≥60	Higher education	Jobless	Bride
K256	M	30	[30-40[	Secondary school	Private sector	Bachelor
K257	M	61	≥60	Analphabet	Private sector	Bride
K258	F	44	[40-50[	Secondary school	Private sector	Bachelor
K259	F	62	≥60	Analphabet	Household	Widow(er)
K260	F	46	[40-50[	Secondary school	Private sector	Bachelor
K261	F	47	[40-50[	Primary school	Household	Bachelor
K262	M	47	[40-50[	Analphabet	Private sector	Bride
K263	F	35	[30-40[	Secondary school	Household	Bachelor
K264	M	45	[40-50[	Primary school	Private sector	Bachelor
K265	F	43	[40-50[	Secondary school	Private sector	Bride
K266	F	46	[40-50[	Secondary school	Private sector	Bride
K267	F	54	[50-60[	Primary school	Private sector	Bride
K268	F	69	≥60	Secondary school	Household	Bride
K269	F	71	≥60	Analphabet	Household	Widow(er)
K270	F	69	≥60	Analphabet	Household	Bride
K271	F	58	[50-60[	Secondary school	Household	Bachelor
K272	F	46	[40-50[	Secondary school	Private sector	Bride
K273	F	52	[50-60[	Secondary school	Private sector	Bachelor
K274	F	39	[30-40[	Secondary school	Household	Bachelor
K275	F	52	[50-60[	Primary school	Private sector	Bride
K276	F	48	[40-50[	Primary school	Private sector	Bachelor
K277	F	53	[50-60[	Secondary school	Household	Bride
K278	F	58	[50-60[	Primary school	Household	Widow(er)
K279	F	45	[40-50[	Secondary school	Private sector	Bride
K280	M	41	[40-50[	Higher education	Private sector	Bride
K281	F	49	[40-50[	Secondary school	Private sector	Bride
K282	F	53	[50-60[	Primary school	Private sector	Bachelor
K283	F	60	≥60	Analphabet	Private sector	Bride

K284	F	46	[40-50[	Secondary school	Household	Bachelor
K285	F	57	[50-60[	Analphabet	Household	Bachelor
K286	M	47	[40-50[	Higher education	Private sector	Bachelor
K287	M	56	[50-60[	Analphabet	Private sector	Bride
K288	F	26	[20-30[	Higher education	Student	Bachelor
K289	M	28	[20-30[	Higher education	Student	Bachelor
K290	F	60	≥60	Analphabet	Private sector	Widow(er)
K291	F	32	[30-40[	Higher education	Private sector	Bachelor
K292	M	27	[20-30[	Higher education	Student	Bachelor
K293	M	42	[40-50[	Analphabet	Private sector	Bride
K294	F	41	[40-50[	Secondary school	Household	Bachelor
K295	M	28	[20-30[	Secondary school	Private sector	Bachelor
K296	F	48	[40-50[	Primary school	Household	Bride
K297	F	68	≥60	Analphabet	Household	Bride
K298	F	64	≥60	Analphabet	Household	Bride
K299	M	59	[50-60[	Secondary school	Private sector	Bride
K300	M	52	[50-60[	Secondary school	Private sector	Bride
K301	M	25	[20-30[	Secondary school	Private sector	Bachelor
K302	F	75	≥60	Analphabet	Jobless	Widow(er)
K303	F	49	[40-50[	Secondary school	Household	Bachelor
K304	F	41	[40-50[	Primary school	Private sector	Bride
K305	F	63	≥60	Secondary school	Jobless	Widow(er)
K306	M	16	<20	Secondary school	Student	Bachelor
K307	F	50	[50-60[	Higher education	Private sector	Bride
D001	M	45	[40-50[	Secondary school	Private sector	Bride
D002	F	64	≥60	Secondary school	Household	Bride

D003	F	52	[50-60[	Secondary school	Household	Bride
D004	M	42	[40-50[	Secondary school	Private sector	Bride
D005	M	69	≥60	Analphabet	Jobless	Bride
D006	F	34	[30-40[	Secondary school	Household	Bride
D007	M	49	[40-50[	Higher education	Private sector	Bride
D008	F	44	[40-50[	Secondary school	Household	Bachelor
D009	M	77	≥60	Analphabet	Jobless	Bride
D010	M	10	<20	Primary school	Student	Bachelor
D011	M	40	[40-50[	Secondary school	Private sector	Bride
D012	M	31	[30-40[	Higher education	Student	Bachelor
D013	F	38	[30-40[	Secondary school	Private sector	Bachelor
D014	F	55	[50-60[	Primary school	Household	Bride
D015	F	41	[40-50[	Secondary school	Private sector	Bachelor
D016	F	20	[20-30[	Secondary school	Student	Bachelor
D017	F	61	≥60	Secondary school	Household	Widow(er)
D018	F	23	[20-30[	Higher education	Student	Bachelor
D019	F	53	[50-60[	Secondary school	Private sector	Bride
D020	F	34	[30-40[	Secondary school	Jobless	Bride
D021	F	36	[30-40[	Higher education	Private sector	Bride
D022	M	10	<20	Primary school	Student	Bachelor
D023	M	28	[20-30[	Secondary school	Private sector	Bachelor
D024	F	22	[20-30[	Secondary school	Student	Bachelor
D025	F	38	[30-40[	Primary school	Household	Bachelor
D026	F	20	[20-30[	Secondary school	Student	Bachelor
D027	M	35	[30-40[	Secondary school	Private sector	Bride
D028	F	40	[40-50[	Higher education	Private sector	Bride
D029	F	53	[50-60[	Primary school	Household	Bride
D030	F	45	[40-50[	Higher education	Private sector	Bride
D031	F	36	[30-40[	Higher education	Household	Bachelor

D032	M	63	≥60	Primary school	Private sector	Bride
D033	F	22	[20-30[	Secondary school	Student	Bachelor
D034	F	38	[30-40[	Higher education	Private sector	Bride
D035	M	57	[50-60[	Secondary school	Private sector	Bride
D036	F	20	[20-30[	Higher education	Student	Bachelor
D037	F	54	[50-60[	Secondary school	Household	Bride
D038	F	27	[20-30[	Higher education	Student	Bachelor
D039	F	25	[20-30[	Secondary school	Household	Bride
D040	F	58	[50-60[	Secondary school	Household	Bride
D041	M	27	[20-30[	Higher education	Student	Bachelor
D042	F	66	≥60	Secondary school	Household	Bride
D043	M	10	<20	Primary school	Student	Bachelor
D044	F	37	[30-40[	Secondary school	Household	Bride
D045	F	78	≥60	Analphabet	Household	Widow(er)
D046	M	63	≥60	Secondary school	Private sector	Bride
D047	M	43	[40-50[	Primary school	Private sector	Bride
D048	F	63	≥60	Primary school	Household	Bride
D049	M	52	[50-60[	Secondary school	Private sector	Bride
D050	F	23	[20-30[	Secondary school	Student	Bachelor
D051	M	42	[40-50[	Secondary school	Private sector	Bride
D052	F	23	[20-30[	Higher education	Student	Bachelor
D053	F	54	[50-60[	Secondary school	Household	Bride
D054	F	31	[30-40[	Secondary school	Household	Bachelor
D055	M	44	[40-50[	Secondary school	Private sector	Bride
D056	M	43	[40-50[	Secondary school	Private sector	Bride
D057	M	40	[40-50[	Secondary school	Private sector	Bride
D058	M	47	[40-50[	Higher education	Private sector	Bride
D059	M	10	<20	Primary school	Student	Bachelor

D060	M	41	[40-50[	Secondary school	Private sector	Bride
D061	M	37	[30-40[	Higher education	Private sector	Bride
D062	F	17	<20	Secondary school	Student	Bachelor
D063	F	30	[30-40[	Secondary school	Household	Bachelor
D064	F	38	[30-40[	Higher education	Private sector	Bachelor
D065	M	30	[30-40[	Secondary school	Private sector	Bachelor
D066	F	33	[30-40[	Secondary school	Household	Bride
D067	F	84	≥60	Analphabet	Household	Widow(er)
D068	F	38	[30-40[	Secondary school	Private sector	Bachelor
D069	M	48	[40-50[	Higher education	Private sector	Bride
D070	F	39	[30-40[	Primary school	Household	Bride
D071	F	44	[40-50[	Higher education	Private sector	Bride
D072	F	34	[30-40[	Secondary school	Private sector	Bride
D073	F	10	<20	Primary school	Student	Bachelor
D074	F	42	[40-50[	Secondary school	Private sector	Bride
D075	F	36	[30-40[	Higher education	Private sector	Bride
D076	M	29	[20-30[	Higher education	Student	Bachelor
D077	F	20	[20-30[	Higher education	Student	Bachelor
D078	M	29	[20-30[	Secondary school	Private sector	Bachelor
D079	F	10	<20	Primary school	Student	Bachelor
D080	M	81	≥60	Analphabet	Jobless	Bride
D081	F	29	[20-30[	Secondary school	Private sector	Bride
D082	F	51	[50-60[	Secondary school	Household	Widow(er)
D083	M	35	[30-40[	Secondary school	Private sector	Bride
D084	F	51	[50-60[	Secondary school	Household	Bride
D085	F	42	[40-50[	Secondary school	Household	Bride
D086	M	64	≥60	Secondary school	Private sector	Bride
D087	M	56	[50-60[	Secondary school	Jobless	Bride
D088	F	33	[30-40[	Secondary school	Household	Bride

D089	M	34	[30-40[	Secondary school	Private sector	Bride
D090	M	58	[50-60[	Secondary school	Private sector	Bride
D091	M	54	[50-60[	Secondary school	Private sector	Bride
D092	M	49	[40-50[	Secondary school	Private sector	Bride
D093	M	48	[40-50[	Secondary school	Private sector	Bride
D094	M	52	[50-60[	Secondary school	Private sector	Bride
D095	F	34	[30-40[	Secondary school	Household	Bride
D096	F	29	[20-30[	Higher education	Private sector	Bachelor
D097	M	25	[20-30[	Higher education	Student	Bachelor
D098	F	55	[50-60[	Secondary school	Private sector	Bride
D099	M	41	[40-50[	Primary school	Private sector	Bachelor
D100	F	48	[40-50[	Secondary school	Household	Bachelor
D101	F	42	[40-50[	Secondary school	Household	Bride
D102	F	45	[40-50[	Secondary school	Private sector	Bride
D103	F	23	[20-30[	Secondary school	Student	Bachelor
D104	M	47	[40-50[	Secondary school	Private sector	Bride
D105	M	15	<20	Secondary school	Student	Bachelor
D106	F	68	≥60	Primary school	Jobless	Bachelor
D107	M	39	[30-40[	Secondary school	Private sector	Bride
D108	M	47	[40-50[	Secondary school	Private sector	Bride
D109	F	36	[30-40[	Secondary school	Private sector	Bachelor
D110	M	45	[40-50[	Secondary school	Private sector	Bride
D111	F	42	[40-50[	Secondary school	Private sector	Widow(er)
D112	F	20	[20-30[	Secondary school	Private sector	Bride
D113	F	23	[20-30[	Secondary school	Private sector	Bride
D114	M	47	[40-50[	Primary school	Private sector	Bride
D115	M	20	[20-30[	Higher education	Private sector	Bride
D116	F	68	≥60	Secondary school	Private sector	Bride

D117	M	39	[30-40[	Primary school	Private sector	Bride
D118	M	47	[40-50[	Secondary school	Private sector	Bride
D119	F	36	[30-40[	Higher education	Private sector	Bride
D120	M	32	[30-40[	Secondary school	Private sector	Bachelor
D121	F	80	≥60	Secondary school	Private sector	Bachelor
D122	F	53	[50-60[	Primary school	Private sector	Bride
D123	F	29	[20-30[	Secondary school	Private sector	Bride
D124	M	66	≥60	Secondary school	Private sector	Bride
D125	M	71	≥60	Secondary school	Private sector	Bride
D126	M	42	[40-50[	Secondary school	Private sector	Bachelor
D127	F	28	[20-30[	Secondary school	Private sector	Bachelor
D128	M	33	[30-40[	Secondary school	Private sector	Bride
D129	F	72	≥60	Primary school	Private sector	Bachelor
D130	F	60	≥60	Secondary school	Private sector	Widow(er)
D131	F	70	≥60	Secondary school	Private sector	Bride
D132	M	29	[20-30[	Secondary school	Private sector	Bride
D133	F	35	[30-40[	Secondary school	Private sector	Bride
D134	M	46	[40-50[	no scolarise	Private sector	Bachelor
D135	M	39	[30-40[	Secondary school	Private sector	Widow(er)
D136	F	65	≥60	Secondary school	Private sector	Bride
D137	M	44	[40-50[	Primary school	Private sector	Bride
D138	F	65	≥60	no scolarise	Private sector	Bride
D139	F	44	[40-50[	Secondary school	Private sector	Widow(er)
D140	F	64	≥60	Secondary school	Private sector	Bride
D141	F	56	[50-60[	Secondary school	Private sector	Bride
D142	F	66	≥60	no scolarise	Private sector	Bride
D143	M	34	[30-40[	Primary school	Private sector	Widow(er)
D144	F	58	[50-60[	Primary school	Private sector	Widow(er)
D145	F	54	[50-60[	Secondary school	Private sector	Bachelor

D146	F	48	[40-50[	Higher education	Private sector	Bachelor
D147	M	52	[50-60[	Secondary school	Private sector	Bride
D148	M	34	[30-40[	Secondary school	Jobless	Bride
D149	F	29	[20-30[	Higher education	Jobless	Bachelor
D150	F	38	[30-40[	Secondary school	Jobless	Bride
D151	F	36	[30-40[	Higher education	Private sector	Bride
D152	M	10	<20	Primary school	Student	Bachelor
D153	M	28	[20-30[	Secondary school	Private sector	Bachelor
D154	F	22	[20-30[	Secondary school	Student	Bachelor
D155	F	38	[30-40[	Primary school	Household	Bachelor
D156	F	20	[20-30[	Secondary school	Student	Bachelor
D157	M	35	[30-40[	Secondary school	Private sector	Bride
D158	F	40	[40-50[	Higher education	Private sector	Bride
D159	F	53	[50-60[	Primary school	Household	Bride
D160	F	45	[40-50[	Higher education	Private sector	Bride
D161	F	36	[30-40[	Higher education	Household	Bachelor
D162	M	63	≥60	Primary school	Private sector	Bride
D163	F	22	[20-30[	Secondary school	Student	Bachelor
D164	F	38	[30-40[	Higher education	Private sector	Bride
D165	M	57	[50-60[	Secondary school	Private sector	Bride
D166	F	20	[20-30[	Higher education	Student	Bachelor
D167	F	54	[50-60[	Secondary school	Household	Bride
D168	F	27	[20-30[	Higher education	Student	Bachelor
D169	F	25	[20-30[	Secondary school	Household	Bride
D170	F	58	[50-60[	Secondary school	Household	Bride
D171	M	27	[20-30[	Higher education	Student	Bachelor
D172	F	66	≥60	Secondary school	Household	Bride
D173	M	10	<20	Primary school	Student	Bachelor
D174	F	37	[30-40[	Secondary school	Household	Bride

D175	F	78	≥60	Analphabet	Household	Widow(er)
D176	M	63	≥60	Secondary school	Private sector	Bride
D177	M	43	[40-50[	Primary school	Private sector	Bride
D178	F	63	≥60	Primary school	Household	Bride
D179	M	52	[50-60[	Secondary school	Private sector	Bride
D180	F	23	[20-30[	Secondary school	Student	Bachelor
D181	M	42	[40-50[	Secondary school	Private sector	Bride
D182	F	23	[20-30[	Higher education	Student	Bachelor
D183	F	54	[50-60[	Secondary school	Household	Bride
D184	F	31	[30-40[	Secondary school	Household	Bachelor
D185	M	44	[40-50[	Secondary school	Private sector	Bride
D186	M	43	[40-50[	Secondary school	Private sector	Bride
D187	M	40	[40-50[	Secondary school	Private sector	Bride
D188	M	47	[40-50[	Higher education	Private sector	Bride
D189	M	10	<20	Primary school	Student	Bachelor
D190	M	41	[40-50[	Secondary school	Private sector	Bride
D191	M	37	[30-40[	Higher education	Private sector	Bride
D192	F	17	<20	Secondary school	Student	Bachelor
D193	F	30	[30-40[	Secondary school	Household	Bachelor
D194	F	38	[30-40[	Higher education	Private sector	Bachelor
D195	M	30	[30-40[	Secondary school	Private sector	Bachelor
D196	F	33	[30-40[	Secondary school	Household	Bride
D197	F	84	≥60	Analphabet	Household	Widow(er)
D198	F	38	[30-40[	Secondary school	Private sector	Bachelor
D199	M	48	[40-50[	Higher education	Private sector	Bride
D200	F	39	[30-40[	Primary school	Household	Bride

Legend: M, male; F, female; code with K, cancer patient; code with D, no-cancer patient

S2. Patients and cancer treatments

Patient code	**Cancer status**	**Location of the pathology**	**Cancer stage**	**Classification**	**Chemotherapy?**
K001	Yes	Cavum	Stage 3	Node	No
K002	Yes	Prostate	Stage 4	Metastasis	No
K003	Yes	Breast	Stage 4	Metastasis	Yes
K004	Yes	Pancreas	Stage 4	Metastasis	Yes
K005	Yes	Colorectal	Stage 4	Metastasis	Yes
K006	Yes	Liver	Stage 4	Metastasis	No
K007	Yes	Cervical	Stage 3	Node	No
K008	Yes	Kaposi sarcoma	Unknown stage		No
K009	Yes	Breast	Stage 3	Node	Yes
K010	Yes	no-Hodgkin lymphoma	Stage 4	Metastasis	No
K011	Yes	Breast	Stage 3	Node	No
K012	Yes	Lung	Stage 4	Metastasis	Yes
K013	Yes	Breast	Stage 4	Metastasis	Yes
K014	Yes	Osteosarcoma	Stage 4	Metastasis	No
K015	Yes	Breast	Stage 3	Node	Yes
K016	Yes	Liver	Unknown stage		No
K017	Yes	Osteosarcoma	Stage 4	Metastasis	Yes
K018	Yes	Lung	Stage 4	Metastasis	Yes
K019	Yes	Cholangiocarcinoma	Stage 4	Metastasis	No
K020	Yes	Cavum	Stage 3	Node	Yes
K021	Yes	Liver	Stage 3	Node	No
K022	Yes	Colorectal	Stage 3	Node	Yes
K023	Yes	Lung	Stage 3	Metastasis	Yes
K024	Yes	Liver	Unknown stage		No

K025	Yes	no-Hodgkin lymphoma	Unknown stage		No
K026	Yes	no-Hodgkin lymphoma	Unknown stage		Yes
K027	Yes	Prostate	Stage 3	Node	No
K028	Yes	Colorectal	Stage 3	Node	No
K029	Yes	Liver	Stage 3	Node	No
K030	Yes	no-Hodgkin lymphoma	Unknown stage		No
K031	Yes	Pancreas	Stage 3	Node	No
K032	Yes	Breast	Stage 4	Metastasis	No
K033	Yes	Breast	Stage 4	Metastasis	No
K034	Yes	Pancreas	Stage 3	Node	No
K035	Yes	Osteosarcoma	Stage 3	Node	Yes
K036	Yes	Breast	Stage 4	Metastasis	Yes
K037	Yes	Breast	Stage 3	Node	Yes
K038	Yes	Cholangiocarcinoma	Stage 4	Metastasis	Yes
K039	Yes	Prostate	Stage 4	Metastasis	No
K040	Yes	Prostate	Stage 4	Metastasis	No
K041	Yes	Prostate	Stage 4	Metastasis	No
K042	Yes	Cervical	Unknown stage		No
K043	Yes	Cervical	Stage 3	Node	No
K044	Yes	Breast	Stage 4	Metastasis	No
K045	Yes	Osteosarcoma	Stage 4	Metastasis	No
K046	Yes	Cervical	Stage 3	Node	Yes
K047	Yes	Kaposi sarcoma	Unknown stage		Yes
K048	Yes	Breast	Stage 3	Node	Yes
K049	Yes	Breast	Stage 4	Metastasis	No
K050	Yes	no-Hodgkin lymphoma	Stage 4	Metastasis	No

K051	Yes	Cholangiocarcinoma	Stage 4	Metastasis	No
K052	Yes	Breast	Stage 4	Metastasis	No
K053	Yes	Cervical	Stage 3	Node	No
K054	Yes	Cervical	Stage 4	Metastasis	No
K055	Yes	Osteosarcoma	Stage 4	Metastasis	Yes
K056	Yes	Breast	Stage 4	Metastasis	Yes
K057	Yes	Lung	Stage 4	Metastasis	Yes
K058	Yes	Breast	Stage 4	Metastasis	Yes
K059	Yes	Colorectal	Stage 3	Node	No
K060	Yes	Breast	Stage 4	Metastasis	Yes
K061	Yes	Breast	Stage 4	Metastasis	No
K062	Yes	Stomach	Stage 3	Node	No
K063	Yes	no-Hodgkin lymphoma	Stage 4	Metastasis	Yes
K064	Yes	Cervical	Stage 4	Metastasis	Yes
K065	Yes	Prostate	Unknown stage		No
K066	Yes	no-Hodgkin lymphoma	Stage 3	Node	Yes
K067	Yes	Liver	Stage 4	Metastasis	No
K068	Yes	Colorectal	Stage 3	Node	Yes
K069	Yes	Liver	Stage 4	Metastasis	No
K070	Yes	Liver	Stage 4	Metastasis	No
K071	Yes	Osteosarcoma	Stage 4	Metastasis	No
K072	Yes	Cervical	Stage 4	Metastasis	No
K073	Yes	no-Hodgkin lymphoma	Stage 4	Metastasis	No
K074	Yes	Colorectal	Stage 4	Metastasis	No
K075	Yes	Breast	Stage 4	Metastasis	Yes
K076	Yes	Breast	Stage 4	Metastasis	No
K077	Yes	Kaposi sarcoma	Stage 4	Metastasis	Yes
K078	Yes	Stomach	Stage 3	Node	No

K079	Yes	Breast	Stage 4	Metastasis	Yes
K080	Yes	Cervical	Unknown stage		Yes
K081	Yes	Cervical	Stage 4	Metastasis	No
K082	Yes	Colorectal	Stage 3	Node	No
K083	Yes	Breast	Stage 3	Node	Yes
K084	Yes	Pancreas	Unknown stage		Yes
K085	Yes	Breast	Stage 4	Metastasis	No
K086	Yes	Prostate	Stage 4	Metastasis	No
K087	Yes	no-Hodgkin lymphoma	Stage 3	Node	Yes
K088	Yes	Breast	Stage 3	Node	Yes
K089	Yes	Prostate	Stage 4	Metastasis	No
K090	Yes	no-Hodgkin lymphoma	Unknown stage		No
K091	Yes	Colorectal	Stage 3	Node	No
K092	Yes	Breast	Stage 3	Node	No
K093	Yes	Cervical	Stage 3	Node	Yes
K094	Yes	Cavum	Unknown stage		No
K095	Yes	Cervical	Unknown stage		Yes
K096	Yes	no-Hodgkin lymphoma	Stage 3	Node	Yes
K097	Yes	Breast	Stage 3	Node	Yes
K098	Yes	no-Hodgkin lymphoma	Unknown stage		Yes
K099	Yes	no-Hodgkin lymphoma	Stage 4	Metastasis	Yes
K100	Yes	Cervical	Stage 4	Metastasis	Yes
K101	Yes	Kaposi sarcoma	Unknown stage		Yes
K102	Yes	Cholangiocarcinoma	Unknown stage		Yes

K103	Yes	Colorectal	Stage 2	Node	Yes
K104	Yes	Colorectal	Stage 4	Metastasis	No
K105	Yes	Breast	Stage 4	Metastasis	Yes
K106	Yes	Breast	Stage 4	Metastasis	Yes
K107	Yes	no-Hodgkin lymphoma	Unknown stage		Yes
K108	Yes	Colorectal	Stage 3	Node	Yes
K109	Yes	Breast	Stage 4	Metastasis	Yes
K110	Yes	Breast	Stage 3	Node	Yes
K111	Yes	Osteosarcoma	Unknown stage		Yes
K112	Yes	Breast	Stage 3	Node	Yes
K113	Yes	Breast	Stage 3	Node	Yes
K114	Yes	Breast	Stage 3	Node	Yes
K115	Yes	Cervical	Stage 3	Node	Yes
K116	Yes	Breast	Stage 3	Node	Yes
K117	Yes	Stomach	Unknown stage		Yes
K118	Yes	Prostate	Stage 4	Metastasis	Yes
K119	Yes	Cervical	Stage 3	Node	Yes
K120	Yes	Stomach	Stage 4	Metastasis	Yes
K121	Yes	Breast	Stage 3	Node	Yes
K122	Yes	no-Hodgkin lymphoma	Unknown stage		Yes
K123	Yes	Breast	Stage 3	Node	Yes
K124	Yes	Stomach	Unknown stage		Yes
K125	Yes	Cervical	Stage 3	Node	Yes
K126	Yes	Breast	Stage 4	Metastasis	Yes
K127	Yes	Breast	Unknown stage		Yes

K128	Yes	Breast	Stage 3	Node	Yes
K129	Yes	Breast	Stage 3	Node	Yes
K130	Yes	Cervical	Stage 4	Metastasis	No
K131	Yes	Breast	Stage 4	Metastasis	No
K132	Yes	Cervical	Stage 4	Metastasis	No
K133	Yes	Breast	Stage 3	Node	No
K134	Yes	Cholangiocarcinoma	Stage 4	Metastasis	No
K135	Yes	Stomach	Stage 4	Metastasis	No
K136	Yes	Colorectal	Stage 4	Metastasis	No
K137	Yes	no-Hodgkin lymphoma	Stage 4	Metastasis	Yes
K138	Yes	Stomach	Stage 3	Node	Yes
K139	Yes	no-Hodgkin lymphoma	Stage 4	Metastasis	Yes
K140	Yes	Pancreas	Stage 4	Metastasis	Yes
K141	Yes	Kaposi sarcoma	Stage 3	Node	Yes
K142	Yes	Cavum	Stage 4	Metastasis	Yes
K143	Yes	Osteosarcoma	Stage 4	Metastasis	Yes
K144	Yes	Kaposi sarcoma	Unknown stage		Yes
K145	Yes	Breast	Stage 4	Metastasis	Yes
K146	Yes	Cervical	Stage 4	Metastasis	No
K147	Yes	Cavum	Stage 4	Metastasis	Yes
K148	Yes	Kaposi sarcoma	Unknown stage		Yes
K149	Yes	Breast	Stage 4	Metastasis	Yes
K150	Yes	Breast	Stage 4	Metastasis	Yes
K151	Yes	Cholangiocarcinoma	Stage 4	Metastasis	No
K152	Yes	Breast	Stage 4	Metastasis	Yes
K153	Yes	Breast	Stage 3	Node	No
K154	Yes	Breast	Stage 4	Metastasis	Yes
K155	Yes	Pancreas	Stage 4	Metastasis	Yes

K156	Yes	Breast	Stage 4	Metastasis	Yes
K157	Yes	Breast	Stage 4	Metastasis	Yes
K158	Yes	no-Hodgkin lymphoma	Stage 4	Metastasis	Yes
K159	Yes	Cavum	Unknown stage		Yes
K160	Yes	Breast	Stage 4	Metastasis	Yes
K161	Yes	Cavum	Unknown stage		Yes
K162	Yes	Cervical	Stage 4	Metastasis	Yes
K163	Yes	Breast	Stage 3	Node	Yes
K164	Yes	Cervical	Stage 4	Metastasis	Yes
K165	Yes	Cavum	Stage 3	Node	Yes
K166	Yes	Prostate	Stage 4	Metastasis	Yes
K167	Yes	Pancreas	Stage 4	Metastasis	No
K168	Yes	Stomach	Unknown stage		Yes
K169	Yes	Stomach	Stage 4	Metastasis	Yes
K170	Yes	Breast	Stage 4	Metastasis	Yes
K171	Yes	Cholangiocarcinoma	Stage 4	Metastasis	No
K172	Yes	Colorectal	Stage 4	Metastasis	No
K173	Yes	Osteosarcoma	Stage 3	Metastasis	Yes
K174	Yes	Colorectal	Stage 4	Metastasis	Yes
K175	Yes	Kaposi sarcoma	Stage 4	Metastasis	No
K176	Yes	Colorectal	Stage 4	Metastasis	No
K177	Yes	Pancreas	Stage 4	Metastasis	No
K178	Yes	Kaposi sarcoma	Stage 3	Node	Yes
K179	Yes	Osteosarcoma	Stage 3	Node	Yes
K180	Yes	Breast	Stage 3	Node	Yes
K181	Yes	no-Hodgkin lymphoma	Unknown stage		Yes

K182	Yes	Lung	Unknown stage		Yes
K183	Yes	no-Hodgkin lymphoma	Stage 2	Tumeur	No
K184	Yes	Prostate	Stage 4	Metastasis	Yes
K185	Yes	Breast	Unknown stage		Yes
K186	Yes	Breast	Stage 3	Node	Yes
K187	Yes	Breast	Stage 4	Metastasis	Yes
K188	Yes	Pancreas	Stage 4	Metastasis	No
K189	Yes	Breast	Stage 4	Metastasis	No
K190	Yes	Breast	Stage 4	Metastasis	No
K191	Yes	Osteosarcoma	Unknown stage		Yes
K192	Yes	Cavum	Unknown stage		Yes
K193	Yes	Colorectal	Stage 4	Metastasis	No
K194	Yes	Breast	Stage 4	Metastasis	Yes
K195	Yes	Kaposi sarcoma	Stage 4	Metastasis	Yes
K196	Yes	Osteosarcoma	Stage 4	Metastasis	Yes
K197	Yes	Osteosarcoma	Stage 4	Metastasis	Yes
K198	Yes	Breast	Stage 4	Metastasis	Yes
K199	Yes	Lung	Stage 4	Metastasis	No
K200	Yes	Breast	Stage 4	Metastasis	Yes
K201	Yes	Colorectal	Stage 4	Metastasis	Yes
K202	Yes	Osteosarcoma	Stage 4	Metastasis	No
K203	Yes	Osteosarcoma	Stage 3	Node	No
K204	Yes	Cervical	Stage 4	Metastasis	Yes
K205	Yes	Cervical	Stage 4	Metastasis	No
K206	Yes	Breast	Stage 3	Node	Yes
K207	Yes	Breast	Stage 3	Node	Yes
K208	Yes	Breast	Stage 4	Metastasis	Yes

K209	Yes	Colorectal	Stage 4	Metastasis	Yes
K210	Yes	Cervical	Stage 3	Node	no
K211	Yes	Breast	Stage 3	Node	Yes
K212	Yes	Cavum	Stage 3	Node	Yes
K213	Yes	Breast	Stage 4	Metastasis	Yes
K214	Yes	Breast	Unknown stage		Yes
K215	Yes	Breast	Stage 4	Metastasis	Yes
K216	Yes	Breast	Unknown stage		Yes
K217	Yes	Cholangiocarcinoma	Stage 4	Metastasis	Yes
K218	Yes	Breast	Stage 4	Metastasis	Yes
K219	Yes	Breast	Stage 3	Node	Yes
K220	Yes	Breast	Stage 4	Metastasis	No
K221	Yes	Breast	Stage 4	Metastasis	No
K222	Yes	Stomach	Stage 4	Metastasis	Yes
K223	Yes	Cervical	Stage 3	Node	No
K224	Yes	no-Hodgkin lymphoma	Stage 4	Metastasis	No
K225	Yes	Stomach	Unknown stage		Yes
K226	Yes	Cervical	Stage 3	Node	Yes
K227	Yes	Cervical	Stage 4	Metastasis	No
K228	Yes	Breast	Stage 4	Metastasis	No
K229	Yes	Esophagus	Unknown stage		Yes
K230	Yes	Cervical	Stage 3	Node	Yes
K231	Yes	Cervical	Stage 4	Metastasis	Yes
K232	Yes	Cervical	Stage 4	Metastasis	Yes
K233	Yes	Osteosarcoma	Stage 4	Metastasis	No
K234	Yes	Breast	Stage 4	Metastasis	No
K235	Yes	Cervical	Stage 4	Metastasis	No

K236	Yes	Prostate	Stage 4	Metastasis	No
K237	Yes	Stomach	Stage 4	Metastasis	No
K238	Yes	Esophagus	Stage 3	Node	No
K239	Yes	Cervical	Stage 4	Metastasis	No
K240	Yes	Stomach	Stage 4	Metastasis	No
K241	Yes	Breast	Stage 4	Metastasis	Yes
K242	Yes	Breast	Stage 3	Node	Yes
K243	Yes	Ovary	Stage 4	Metastasis	Yes
K244	Yes	Breast	Stage 3	Node	Yes
K245	Yes	Cervical	Stage 4	Metastasis	Yes
K246	Yes	Ovary	Stage 4	Metastasis	Yes
K247	Yes	Breast	Stage 4	Metastasis	No
K248	Yes	Liver	Stage 4	Metastasis	No
K249	Yes	Pancreas	Unknown stage		Yes
K250	Yes	Cervical	Stage 4	Metastasis	no
K251	Yes	Ovary	Stage 4	Metastasis	Yes
K252	Yes	Osteosarcoma	Stage 4	Metastasis	Yes
K253	Yes	Esophagus	Stage 4	Metastasis	No
K254	Yes	Stomach	Stage 4	Metastasis	Yes
K255	Yes	Prostate	Stage 4	Metastasis	Yes
K256	Yes	Liver	Stage 4	Metastasis	No
K257	Yes	Liver	Stage 4	Metastasis	No
K258	Yes	Cervical	Stage 4	Metastasis	No
K259	Yes	Ovary	Stage 4	Metastasis	Yes
K260	Yes	Breast	Stage 3	Node	Yes
K261	Yes	Cervical	Unknown stage		Yes
K262	Yes	Esophagus	Stage 3	Node	Yes
K263	Yes	Cervical	Stage 3	Node	Yes

K264	Yes	Esophagus	Unknown stage		No
K265	Yes	Breast	Stage 3	Node	Yes
K266	Yes	Breast	Stage 4	Metastasis	Yes
K267	Yes	Kaposi sarcoma	Unknown stage		Yes
K268	Yes	Breast	Stage 4	Metastasis	Yes
K269	Yes	Breast	Stage 4	Metastasis	Yes
K270	Yes	Pancreas	Stage 4	Metastasis	Yes
K271	Yes	Breast	Stage 3	Node	Yes
K272	Yes	Cervical	Stage 4	Metastasis	No
K273	Yes	Breast	Stage 4	Metastasis	No
K274	Yes	Cervical	Stage 4	Metastasis	Yes
K275	Yes	Breast	Stage 4	Metastasis	Yes
K276	Yes	Breast	Stage 4	Metastasis	Yes
K277	Yes	Breast	Stage 4	Metastasis	Yes
K278	Yes	Lung	Stage 3	Node	Yes
K279	Yes	Breast	Stage 4	Metastasis	Yes
K280	Yes	Liver	Stage 4	Metastasis	No
K281	Yes	Breast	Stage 3	Node	Yes
K282	Yes	Breast	Stage 4	Metastasis	No
K283	Yes	Kaposi sarcoma	Stage 4	Metastasis	No
K284	Yes	Cervical	Stage 4	Metastasis	No
K285	Yes	Cervical	Stage 4	Metastasis	No
K286	Yes	Liver	Stage 4	Metastasis	No
K287	Yes	Stomach	Stage 4	Metastasis	Yes
K288	Yes	Breast	Unknown stage		Yes
K289	Yes	Lung	Stage 3	Node	Yes
K290	Yes	Pancreas	Stage 4	Metastasis	Yes

K291	Yes	Colorectal	Stage 3	Node	Yes
K292	Yes	Kaposi sarcoma	Stage 4	Metastasis	Yes
K293	Yes	Breast	Stage 4	Metastasis	No
K294	Yes	Cervical	Stage 4	Metastasis	Yes
K295	Yes	Kaposi sarcoma	Stage 3	Node	Yes
K296	Yes	Breast	Stage 3	Node	Yes
K297	Yes	Pancreas	Stage 4	Metastasis	Yes
K298	Yes	Breast	Unknown stage		Yes
K299	Yes	Pancreas	Stage 4	Metastasis	No
K300	Yes	Prostate	Stage 4	Metastasis	No
K301	Yes	Osteosarcoma	Stage 4	Metastasis	No
K302	Yes	Liver	Stage 4	Metastasis	No
K303	Yes	Ovary	Stage 4	Metastasis	No
K304	Yes	Ovary	Stage 4	Metastasis	No
K305	Yes	Breast	Stage 4	Metastasis	No
K306	Yes	no-Hodgkin lymphoma	Stage 4	Metastasis	No
K307	Yes	Breast	Stage 4	Metastasis	No

S3. Patients with *K. pneumoniae infection and antibiogram*

Patient code	Antibiotics and susceptibility profile																				
	IMP	AMX	CAZ	FOX	CTX	CXM	COT	ERY	AMK	GEN	CIP	OFX	NAL	CTR	COL	PRL	TET	VAN	NIT	ATM	AMC	FOS
K033	S	R	R	R	R	R	R	R	S	S	R	R	R	R	S	R	R	R	S	R	R	R
K040	S	R	S	S	S	S	S	S	S	S	S	S	R	R	R	R	S	R	S	S	S	S
K048	S	R	R	R	R	R	S	R	S	S	S	S	S	S	R	R	R	R	S	S	S	S
K067	S	R	R	R	R	R	S	R	S	S	S	S	S	R	R	R	S	R	R	S	R	R
K106	S	I	I	R	R	R	S	R	S	S	R	R	R	R	R	R	R	R	R	R	R	R
K118	S	R	R	R	R	R	R	R	S	S	R	R	S	R	S	R	R	R	S	R	R	R
K120	S	R	I	S	R	R	S	R	S	S	I	R	R	R	R	R	R	R	S	S	R	R
K165	S	R	R	R	R	R	R	R	S	S	S	S	S	R	S	R	R	R	R	S	S	S
K175	S	R	R	R	R	R	R	R	S	S	R	R	R	R	R	R	R	R	R	R	R	S
K176	S	R	R	R	R	R	S	R	S	S	R	R	S	R	R	R	R	R	S	S	R	S
K177	S	R	R	R	R	R	S	R	S	S	R	R	R	R	S	R	R	R	S	R	S	S
K195	S	R	R	R	R	R	R	S	S	S	S	S	S	R	R	R	R	R	S	S	R	S
K197	S	R	R	R	S	R	S	R	S	S	S	S	R	R	R	R	R	R	R	S	S	S
K203	S	R	R	R	R	R	S	R	S	S	R	R	R	R	R	R	R	R	S	R	R	R
K205	S	R	R	S	R	R	R	R	S	S	S	S	R	S	S	R	R	R	S	R	R	S
K216	S	R	S	R	R	R	S	S	S	S	S	S	S	S	S	R	S	R	R	S	S	S
K241	S	R	R	R	R	R	S	R	S	S	S	S	S	R	R	R	R	R	R	S	R	S
K254	S	S	S	S	S	S	S	S	S	S	S	S	S	S	S	R	R	R	S	S	S	S
K257	S	R	R	R	R	R	S	R	S	S	R	R	S	S	R	R	R	R	S	S	S	S
K299	S	R	R	R	R	R	R	R	S	S	R	R	R	R	S	R	R	R	R	S	R	S
D006	R	R	R	S	R	R	R		S	S	R	R	R	R	R	R	R	R	R	R		
D070	S	R	S	S	S	S	S	S	S	S	S	S	R	S	S	R	S	S	S	S	S	S
D071	S	R	S	S	S	S	S	S	R	S	R	R	R	S	S	R	R	S	S	S	R	S
D077	S	R	R	R	R	R	S	R	S	S	S	S	S	S	S	R	S	S	S	S	S	S

D081	S	R	S	R	R	R	R	S	S	S	I	I	R	S	S	R	S	S	S	S	S	S
D093	S	S	S	S	S	S	S	S	S	S	S	S	S	S	S	S	S	S	S	S	S	S
D099	S	R	R	R	R	R	R	R	S	S	R	R	R	R	S	R	R	R	R	R	R	S
D109	S	R	R	R	R	R	S	R	S	S	R	R	R	R	S	R	R	R	R	R	R	S
D147	S	R	S	S	S	S	S	S	S	S	S	S	R	S	S	R	S	S	S	S	S	S
D178	S	R	S	S	S	S	S	S	S	S	S	S	R	S	S	R	S	S	S	S	S	S

Legend: M, male; F, female; code with K, cancer patient; code with D, non-cancer patient

S4. Patients with *K. oxytoca infection and antibiogram*

Patients code	Antibiotics and susceptibility profile																					
	IMP	AMX	CAZ	FOX	CTX	CXM	COT	ERY	AMK	GEN	CIP	OFX	NAL	CTR	COL	PRL	TET	VAN	NIT	ATM	AMC	FOS
K010	S	R	R	R	R	R	R	R	S	S	R	R	S	S	S	R	R	R	R	I	R	R
K014	R	R	R	R	R	R	S	R	S	S	R	R	S	R	R	R	R	R	R	R	R	R
K019	S	R	R	R	R	R	S	S	S	S	R	R	R	R	R	R	R	R	S	S	R	R
K023	S	R	R	S	R	R	S	S	S	S	R	R	R	R	R	R	R	R	S	S	R	R
K031	S	R	R	S	R	R	R	R	S	S	R	R	R	R	R	R	R	R	R	S	R	S
K055	S	R	R	R	R	R	S	R	S	S	R	R	R	R	S	R	R	R	S	S	S	S
K060	S	R	R	R	R	R	R	R	S	S	R	R	R		S	R	R	R	S	S	S	S
K081	S	R	R	S	S	S	R	R	S	S	R	R	S	R	S	R	R	R	R	R	S	R
K096	S	R	R	R	R	R	S	R	S	S	R	R	R	R	R	R	R	R	R	R	R	R
K107	R	R	R	R	R	R	R	R	S	S	R	R	R	R	S	R	R	R	S	R	R	R
K121	S	S	S	S	S	S	S	R	S	S	R	R	R	R	R	R	R	R	R	R	R	R
K132	S	R	S	S	S	S	S	S	S	S	R	R	R	S	S	R	R	R	S	S	S	S
K136	S	R	S	S	S	S	S	S	S	S	S	S	S	S	R	R	R	R	R	R	R	S
K140	S	R	R	R	S	R	S	R	S	S	S	S	S	R	R	R	R	R	S	R	R	S
K143	S	R	R	R	R	R	R	R	R	S	S	S	S	R	R	R	R	R	R	R	R	R
K152	S	S	R	R	R	R	S	R	S	S	S	S	S	R	R	R	R	R	S	S	S	S
K155	S	S	S	R	S	S	S	S	S	S	S	S	S	S	S	R	S	S	S	S	S	S
K159	S	R	R	R	R	R	R	R	R	S	R	R	R	R	S	R	R	R	R	R	R	R
K164	S	R	R	R	R	R	R	R	S	S	R	R	R	R	S	R	R	R	S	R	R	R
K179	S	S	R	R	S	R	S	R	S	S	R	R	R	S	S	R	R	R	R	R	R	S
K187	S	R	S	R	R	R	S	R	S	S	S	S	S	S	S	S	S	S	S	S	R	S
K192	S	R	R	R	S	R	S	R	S	S	R	R	R	R	R	R	R	R	S	R	R	S
K194	S	R	S	S	S	S	R	R	S	S	S	S	S	S	R	R	R	R	S	R	S	S
K195	S	R	S	S	S	S	R	R	S	S	S	S	S	S	R	R	R	R	S	R	S	S

K210	S	R	R	R	R	R	S	R	S	S	S	S	R	S	S	R	S	R	S	S	S	S
K219	S	R	S	S	R	R	S	R	S	S	S	S	S	S	S	R	S	R	S	S	S	S
K237	S	R	R	R	R	R	R	R	S	S	R	R	R	R	R	R	R	R	R	S	S	S
K248	S	R	R	R	R	R	S	R	S	S	S	S	R	R	R	R	R	R	R	R	R	R
K249	S	R	R	R	R	R	R	R	R	S	S	S	R	R	R	R	R	R	R	R	R	R
K252	S	R	R	R	R	R	S	R	S	S	R	R	R	R	S	R	R	R	R	S	R	S
K256	S	R	R	R	R	R	S	R	S	S	R	R	R	R	R	R	R	R	S	R	R	R
K279	S	R	R	R	R	R	S	R	S	S	S	S	R	S	S	S	S	S	S	S	S	S
K280	S	R	R	R	R	R	S	R	S	S	R	R	R	S	R	R	R	R	R	S	S	S
K281	S	R	R	R	S	R	S	R	S	S	R	R	R	S	R	R	R	R	R	S	R	S
K284	S	S	R	S	S	S	S	S	S	S	S	S	S	S	S	R	S	R	R	S	S	S
K286	S	R	S	R	R	R	R	R	S	S	R	R	R	R	S	R	R	R	R	R	R	R
K292	S	R	R	R	R	R	S	R	S	S	S	S	R	R	R	R	R	R	R	R	R	R
D002	S	R	S	R	R	R	R	R	R	R	R	R	R	S	R	R	S	R	S	R	S	S
D004	S	R	S	R	R	R	S	S	S	S	S	S	R	R	S	R	R	S	S	S	R	S
D008	S	R	S	S	S	S	S	S	S	S	S	S	S	S	S	S	R	S	S	S	S	S
D014	S	R	S	R	S	S	S	S	S	S	R	R	R	S	R	R	R	S	S	S	R	S
D021	S	R	S	R	R	R	S		S	R	S	S	S		S	R	S	R	S	R		
D027	S	S	S	S	S	S	R	S	S	S	S	S	R	S	S	S	S	S	S	S	S	S
D031	S	S	S	S	S	S	S	S	S	S	S	S	S	S	S	S	S	S	S	S	S	S
D036	S	R	S	S	S	S	S	S	S	S	R	R	R	S	S	S	R	R	S	S	S	S
D037	S	R	R	R	R	R	R	R	S	S	R	R	R	R	S	R	R	R	S	R	R	S
D039	S	R	S	S	S	S	S	S	S	S	R	R	R	R	S	R	R	R	S	S	R	S
D053	S	R	S	S	S	S	S	S	S	S	S	S	R	S	S	R	R	S	S	R	S	S
D054	S	R	S	S	S	S	S	S	S	S	S	S	R	S	S	S	S	S	S	S	S	S
D056	S	R	S	S	S	S	R	S	S	S	S	S	S	R	R	S	S	S	S	S	S	R
D068	S	R	S	R	R	R	S	S	S	S	R	R	R	R	R	R	R	R	R	S	S	R
D087	S	R	R	R	R	R	S	S	S	S	S	S	S	S	S	R	S	S	S	R	R	S
D091	S	S	S	S	S	S	S	S	S	S	S	S	S	S	S	S	S	S	S	S	S	S

D092	S	S	S	S	S	S	S	S	S	S	R	R	S	S	S	S	R	S	S	S	S	S
D095	S	R	R	R	R	R	R	R	S	S	I	I	R	R	R	R	R	R	R	R	R	I
D096	S	R	R	R	R	R	R	R	S	S	R	R	R	R	S	R	R	R	R	S	R	R
D102	S	S	S	S	S	S	S	S	S	S	R	R	S	S	S	S	R	S	S	S	S	S
D103	S	R	S	S	S	S	S	S	S	S	S	S	R	S	R	R	R	S	S	R	S	S
D151	S	R	S	S	S	S	R	S	S	S	S	S	S	R	R	S	S	S	S	S	S	R
D174	S	R	S	S	S	S	R	S	S	S	S	S	S	R	R	S	S	S	S	S	S	R

S5. Patients with *P. mirabilis infection and antibiogram*

Patient code	Antibiotics and susceptibility profile																				
	IMP	AMX	CAZ	FOX	CTX	CXM	COT	ERY	AMK	GEN	CIP	OFX	NAL	CTR	COL	PRL	TET	VAN	NIT	ATM	AMC	FOS
K031	S	R	R	R	R	R	S	S	S	S	R	R	R	R	R	R	R	R	S	S	R	S
K039	S	R	S	S	S	R	S	S	S	S	R	R	R	R	R	R	R	R	S	S	R	R
K042	S	R	R	S	R	R	S	S	S	S	R	R	R	R	R	R	R	R	S	S	R	R
K060	S	R	S	R	S	S	S	S	S	S	S	S	S	S	R	R	R	R	S	S	S	S
K065	S	S	S	S	S	S	S	S	S	S	S	S	S	R	R	S	S	R	S	S	S	S
K066	S	S	S	S	S	S	S	S	S	S	S	S	S	R	R	S	R	R	S	S	S	S
K084	S	R	I	R	R	R	S	R	S	S	R	R	R	R	R	R	R	R	S	S	S	S
K090	S	R	R	R	R	R	R	R	S	S	R	R	R	S	R	R	R	R	S	S	R	R
K115	S	R	R	S	R	R	S	R	S	S	R	R	R	R	S	R	R	R	S	S	R	R
K126	S	R	R	R	R	R	R	R	S	S	R	R	R	R	R	R	R	R	R	R	R	S
K129	S	R	R	R	S	R	S	R	S	S	S	S	S	S	R	R	S	R	R	R	R	R
K136	S	R	S	S	S	S	S	S	S	S	S	S	S	S	S	R	R	S	S	S	S	S
K138	S	R	R	S	S	R	R	R	S	R	R	R	R	S	R	R	R	R	S	R	R	S
K157	S	R	R	R	S	R	S	R	S	S	S	S	R	S	S	R	R	R	R	R	R	R
K177	S	R	R	R	S	R	S	R	S	S	R	R	R	S	S	R	R	R	R	R	R	R
K211	S	S	R	R	R	R	R	R	S	S	S	S	S	R	R	R	R	R	R	R	R	R
K231	S	R	R	R	R	R	R	R	S	S	S	R	R	R	R	R	R	R	R	R	R	S
K272	S	R	R	R	S	R	S	R	S	S	R	R	R	S	S	R	R	R	R	R	R	R
D003	S	R	S	R	R	R	R	R	S	S	R	R	R	R	R	R	R	R	R	R	R	S
D013	S	R	S	S	S	S	S		S	S	S	S	S	S	S	R	S	R	S	S		
D024	S	R	S	S	R	R	S		S	S	S	S	S	S	R	R	S	R	S	S	R	R
D045	S	R	S	R	R	R	R	R	S	S	R	R	S	S	S	R	R	R	S	S	R	R
D062	S	R	R	R	S	S	S	S	S	S	I	R	S	R	R	R	R	R	S	S	S	S
D064	S	R	S	S	S	S	S	S	R	S	S	S	S		R	R	S	R	S	S	S	S
D065	S	S	S	S	S	S	S	S	S	S	S	S	S	S	S	S	S	S	S	S	S	S

D067	S	R	R	R	S	S	S	S	R	S	S	S	S	S	S	S	S	R	S	S	S	S
D070	S	R	S	R	R	R	R	R	S	S	R	R	R	R	S	R	R	R	S	S	R	R
D076	S	R	S	R	R	R	R	R	S	S	R	R	R	R	R	R	R	R	S	R	R	S
D085	R	R	R	R	R	R	S		S	S	S	S	S		R	R	S	R	R	I	S	S
D091	S	R	S	S	S	S	S	S	S	S	S	S	R	S	S	R	R	S	S	S	S	S
D105	S	R	R	R	R	R	S	R	R	R	R	R	R	R	R	R	R	R	R	R	S	R
D108	S	R	R	R	R	R	S	R	S	S	R	S	S	R	S	R	R	R	R	R	S	R

S6. Patients with *P. vulgaris infection and antibiogram*

Patient code	Antibiotics and susceptibility profile																				
	IMP	AMX	CAZ	FOX	CTX	CXM	COT	ERY	AMK	GEN	CIP	OFX	NAL	CTR	COL	PRL	TET	VAN	NIT	ATM	AMC	FOS
K009	S	R	R	R	R	R	I	S	S	S	R	S	R	R	S	R	R	R	S	S	R	S
K010	S	R	R	R	R	R	S	R	S	S	R	R	R	R	R	R	R	R	R	R	R	R
K014	S	R	R	R	R	R	R	R	S	R	R	R	R	R	R	R	R	R	R	R	R	R
K015	S	R	S	S	S	S	S	S	S	S	S	S	S	S	R	R	R	R	S	S	S	S
K023	S	R	R	R	R	R	S	R	S	S	R	R	R	R	R	R	R	R	R	R	R	R
K024	S	R	R	R	R	R	R	R	S	S	R	R	R	R	R	R	R	R	S	R	R	R
K032	S	R	R	R	R	R	R	R	S	S	R	R	R	R	R	R	R	R	R	R	R	R
K058	S	R	S	S	R	S	R	R	S	S	S	S	S	S	R	R	R	R	S	S	R	R
K067	S	R	R	S	R	R	S	R	S	S	S	S	S	S	R	R	R	R	R	R	R	S
K097	S	R	S	S	S	S	S	R	S	S	S	S	S	S	R	R	R	R	S	S	R	S
K109	S	R	R	S	R	R	R	R	S	S	S	S	R	S	R	R	R	R	R	R	R	S
K125	S	R	S	S	S	S	S	R	S	S	R	S	S	R	R	R	R	R	R	R	S	S
K130	S	R	R	S	R	R	S	R	R	R	R	R	R	R	R	R	R	R	R	R	R	R
K131	S	R	R	R	R	R	S	R	S	S	R	R	R	R	S	R	R	R	R	S	R	S
K134	S	R	R	R	R	R	S	R	S	S	R	S	R	R	R	R	R	R	S	S	R	S
K139	S	S	R	R	S	R	S	R	S	S	S	S	S	R	S	R	R	R	R	R	R	S
K141	S	R	R	R	R	R	S	R	R	S	S	S	S	R	S	R	R	R	S	S	R	S
K143	S	R	R	R	R	R	R	R	R	S	R	R	R	R	R	R	R	R	R	R	R	R
K147	S	R	R	R	R	R	R	R	R	R	R	R	R	R	R	R	R	R	R	R	R	R
K152	S	S	R	R	R	R	R	R	R	S	S	S	S	R	R	R	R	R	S	S	R	R
K165	S	R	R	R	R	R	R	R	S	S	S	S	R	R	S	R	R	R	R	R	R	S
K176	S	R	R	R	R	R	R	R	R	R	R	R	R	R	R	R	R	R	R	R	R	R
K177	S	R	R	R	R	R	R	R	S	S	R	R	R	R	R	R	R	R	S	R	R	R
K178	S	S	S	S	S	S	S	S	S	S	S	S	S	S	R	R	R	R	R	S	R	S
K188	S	R	R	R	R	R	S	R	S	S	S	S	R	R	S	R	R	R	R	S	R	S

K189	S	R	R	R	R	R	S	R	S	S	R	R	R	R	S	R	R	R	R	S	R	S
K190	S	R	R	R	R	R	S	R	S	S	R	R	R	S	R	R	R	R	S	S	R	S
K192	S	R	R	R	S	R	S	S	S	S	S	S	R	S	R	R	R	R	R	S	R	R
K206	S	R	R	R	R	R	S	R	S	S	R	R	R	S	S	R	R	R	R	R	R	S
K207	S	R	R	R	R	R	S	R	S	S	R	R	R	S	R	R	R	R	S	S	R	S
K209	S	R	R	S	R	R	S	R	S	S	R	R	R	R	R	R	R	R	R	R	R	R
K215	S	R	R	R	R	R	S	R	S	S	R	R	R	R	S	R	R	R	R	R	R	R
K224	S	R	S	R	S	R	R	R	S	S	S	S	S	S	S	R	R	R	S	S	R	S
K225	S	R	R	R	R	R	S	R	S	S	R	R	R	R	R	R	S	R	R	R	S	S
K233	S	S	S	S	S	S	S	S	S	S	S	S	R	S	S	R	S	S	S	S	S	S
K238	S	R	R	R	R	R	S	R	S	S	R	R	R	S	R	R	R	R	R	S	R	S
K240	S	R	R	R	R	R	S	R	S	S	S	S	S	R	R	R	R	R	R	R	R	S
K242	S	R	R	R	R	R	R	R	S	S	R	R	R	S	S	R	R	R	S	S	R	S
K249	S	R	R	R	R	R	R	R	S	S	R	R	R	R	R	R	R	R	R	R	R	S
K266	S	R	R	R	R	R	S	R	S	S	R	R	R	S	R	R	R	R	S	S	R	S
K274	S	R	R	R	R	R	S	R	S	S	R	R	R	S	S	R	R	R	R	S	S	S
K282	S	R	R	R	R	R	R	R	S	R	R	R	R	R	R	R	R	R	R	R	R	R
K293	S	R	R	R	R	R	R	R	S	S	R	R	R	R	R	R	R	R	S	R	R	R
K294	S	R	R	R	R	R	R	R	S	S	R	R	R	R	R	R	R	R	R	R	R	R
K301	S	R	R	R	R	R	S	R	S	S	S	S	S	S	S	R	S	R	S	S	R	S
K304	S	R	R	R	R	S	R	R	S	S	R	R	R	R	R	R	R	R	R	R	R	S
D010	S	R	S	S	R	S	R	S	S	S	R	R	R	R	S	R	R	R	S	S	R	S
D012	S	S	S	S	S	S	S	S	S	S	S	S	S	S	S	S	S	S	S	S	S	S
D013	S	R	S	S	S	S	S	S	S	S	S	S	R	S	S	R	R	S	S	R	S	S
D014	S	S	S	S	S	S	S	S	S	S	S	S	S	S	S	S	S	S	S	S	S	S
D015	S	R	S	R	R	R	R	R	S	S	R	R	R	R	R	R	R	R	R	S	R	S
D017	S	R	R	S	R	R	S	I	I	S	R	R	R	S	S	R	R	R	S	S	R	S
D018	S	S	S	S	S	S	R	S	S	S	S	S	S	S	R	S	S	S	S	S	S	S
D021	S	S	S	S	S	S	S	S	S	S	S	S	S	S	S	S	R	S	S	S	S	S

D022	S	R	R	R	R	R	S	S	S	S	R	R	R	R	R	R	R	R	S	R	S	S
D026	S	R	S	R	S	R	R	S	S	S	S	S	R	R	S	R	R	R	S	S	S	S
D028	S	R	R	R	R	R	R	R	S	S	R	R	R	R	R	R	R	R	R	R	R	S
D031	S	R	R	R	R	R	R	R	R	S	R	R	R	R	R	R	R	R	R	R	R	S
D032	S	R	R	R	R	R	R	R	S	S	R	R	R	R	R	R	R	R	S	S	R	S
D033	S	R	S	S	S	S	S	S	S	S	R	R	R	S	S	R	S	S	S	S	S	S
D041	S	R	S	S	S	S	S	S	S	S	S	S	R	S	S	R	R	S	S	S	S	S
D042	S	S	S	S	S	S	S	S	S	S	R	R	R	S	S	S	S	S	S	S	S	S
D043	S	R	R	R	R	R	R	R	S	S	R	R	R	R	S	R	R	R	S	R	R	S
D044	S	R	S	S	S	S	R	S	S	S	S	S	S	S	S	S	R	S	S	S	S	S
D046	S	R	S	S	S	S	S	S	S	S	S	S	S	S	S	R	S	S	S	S	R	S
D047	S	R	R	R	R	S	R	R	S	S	S	S	S	R	S	R	R	R	S	S	R	S
D054	S	R	S	S	S	S	S	S	S	S	S	S	S	S	S	R	R	S	S	S	R	S
D055	S	R	R	R	R	R	R	R	S	S	R	R	R	R	R	R	R	R	R	R	R	R
D056	S	R	S	S	S	S	R	S	S	S	S	S	S	S	S	R	S	S	S	S	S	S
D057	S	R	S	S	S	S	S	S	S	S	I	I	R	S	S	R	S	R	S	S	S	S
D059	S	R	S	R	R	S	S	S	S	S	S	S	R	R	R	R	R	R	S	S	S	S
D061	R	R	R	S	S	I	S	R	S	S	S	S	S	S	S	S	R	R	S	R	S	S
D063	S	S	S	S	S	S	S	S	S	S	S	S	R	S	S	S	S	S	S	R	S	S
D066	S	S	S	R	R	R	S	S	S	S	S	S	R	R	R	S	R	S	S	S	S	R
D069	S	R	S	S	S	S	S	S	S	S	S	S	S		S	S	S	R	S	I	S	S
D074	S	R	R	R	R	R	S	R	S	S	R	R	R	R	R	R	R	R	R	R	R	S
D085	S	R	S	S	S	S	S	S	S	S	S	S	S	S	S	R	S	S	S	S	S	S
D088	S	R	S	R	S	S	S	R	R	S	R	R	R	R	R	R	R	R	R	S	R	S
D089	S	S	S	S	S	S	S	S	S	S	S	S	S	S	S	S	S	S	S	S	S	S
D093	S	S	S	S	S	S	S	S	S	S	S	S	R	S	S	S	S	S	S	S	R	S
D094	S	S	S	S	S	S	S	S	S	S	S	S	S	S	S	S	S	S	S	S	S	S
D097	S	R	S	R	R	R	S	R	S	S	I	I	R	S	S	R	R	R	R	S	R	S

D101	S	R	R	S	S	S	S	S	R	R	S	I	S		S	S	R	R	S	I	S	S
D102	S	R	S	S	S	S	I	R	R	S	R	R	R		R	R	R	R	R	R	R	R
D104	S	S	S	S	S	S	S	S	S	S	S	S	S	S	S	S	S	S	S	S	S	S
D109	S	R	R	R	R	R	R	R	R	S	S	I	S		S	R	R	R	S	R	S	R
D137	S	R	S	S	S	S	S	S	S	S	S	S	R	S	S	S	S	S	S	S	S	S
D149	S	R	S	S	S	S	R	S	S	S	S	S	S	S	S	R	R	S	S	S	R	S
D159	S	R	S	S	S	S	R	S	S	S	R	R	S	S	S	R	R	R	S	S	R	R
D164	S	R	R	R	R	R	S	R	S	S	R	R	R	R	R	R	R	R	R	S	R	S
D170	S	R	S	R	R	S	S	S	S	S	S	S	R	R	R	R	R	R	S	S	S	S
D176	S	R	S	S	S	S	S	S	S	S	S	S	S	S	S	R	S	S	S	S	S	S

S7. Patients with *E. cloacae infection and antibiogram*

Patient code	Antibiotics and susceptibility profile																				
	IMP	AMX	CAZ	FOX	CTX	CXM	COT	ERY	AMK	GEN	CIP	OFX	NAL	CTR	COL	PRL	TET	VAN	NIT	ATM	AMC	FOS
K013	S	R	S	S	S	S	R	S	S	S	S	S	R	R	R	R	R	R	S	S	R	S
K032	S	R	R	R	R	R	R	R	S	S	R	R	R	R	R	R	R	R	R	R	R	S
K035	S	R	R	R	R	R	S	S	S	S	R	R	R	R	R	R	R	R	S	S	R	S
K081	S	R	S	S	S	R	S	R	S	S	S	S	R		R	R	R	R	S	S	R	R
K110	S	S	S	S	S	S	S	R	S	S	S	S	S	S	S	R	S	R	S	S	S	S
K111	S	R	S	S	S	S	S	R	S	S	R	R	R	S	R	R	R	R	S	R	R	R
K224	S	R	R	R	R	R	R	R	S	S	R	R	R	R	R	R	R	R	R	R	R	R
K286	S	R	R	R	R	R	S	R	S	S	R	R	R	R	R	R	R	R	S	R	R	R
D058	S	R	S	S	S	S	S	S	S	S	R	R	R	R	R	R	R	R	S	S	R	S
D092	S	S	S	S	S	S	S	S	S	S	S	S	S	S	S	R	R	R	S	S	S	S

S8. Patients with *Y. intermedia infection and antibiogram*

Patient code	Antibiotics and susceptibility profile																				
	IMP	AMX	CAZ	FOX	CTX	CXM	COT	ERY	AMK	GEN	CIP	OFX	NAL	CTR	COL	PRL	TET	VAN	NIT	ATM	AMC	FOS
K027	S	R	S	S	S	R	R	R	S	R	R	R	R	R	R	R	R	R	R	R	R	S
K095	S	R	R	R	R	R	S	R	S	S	S	S	R	S	R	R	R	R	S	R	R	S
K102	S	R	R	R	R	R	R	R	S	R	R	R	R	S	R	R	R	R	R	R	R	R
K107	S	R	R	S	R	R	R	R	S	S	R	R	R	R	R	R	R	R	R	R	R	R
K216	S	R	R	R	R	R	S	R	S	S	S	S	R	R	R	R	R	R	R	R	R	S

S9. Patients with *S. typhi infection and antibiogram*

Patient code	Antibiotics and susceptibility profile																				
	IMP	AMX	CAZ	FOX	CTX	CXM	COT	ERY	AMK	GEN	CIP	OFX	NAL	COL	PRL	TET	VAN	NIT	CTR	ATM	AMC	FOS
K055	S	R	R	S	R	R	S	S	S	S	S	S	R	R	R	R	R	S	S	S	S	R
K081	S	R	R	R	S	R	S	R	S	S	S	S	S	R	R	S	R	S	S	S	R	S
K097	S	R	R	S	R	R	R	R	S	S	R	R	R	R	R	R	R	S	R	R	R	R
K145	S	R	R	R	S	R	S	R	S	S	S	S	S	S	R	R	R	R	S	S	R	S
K192	R	R	R	R	R	R	R	R	S	S	S	S	R	R	R	R	R	R	R	R	R	R
K216	S	R	R	R	R	R	S	R	S	S	R	R	R	R	R	R	R	R	R	R	R	R
K229	S	R	R	R	R	R	R	R	S	S	R	R	R	R	R	R	R	R	R	R	R	R
K230	S	R	S	R	R	R	R	R	S	S	S	S	R	S	R	R	R	R	S	S	S	S
K274	S	R	R	R	S	R	R	R	S	S	R	R	R	S	R	R	R	R	S	S	S	S
K287	S	R	R	R	R	R	S	R	S	S	R	R	R	R	R	S	R	R	R	R	R	R
K291	S	R	R	R	R	R	S	R	S	S	R	R	R	R	R	R	R	R	R	R	R	S
K296	S	R	S	R	S	R	R	R	R	S	S	S	R	R	R	R	R	R	S	S	S	S
K300	S	R	R	R	R	R	R	R	R	S	R	R	R	S	R	R	R	R	R	R	R	R
D058	S	R	R	R	R	R	R	R	S	S	R	R	R	S	R	R	R	S	R	R	R	R
D061	S	R	S	S	R	R	R	R	S	S	R	R	R	S	R	R	R	S	R	S	S	S
D063	S	R	S	S	R	R	R	R	S	S	R	R	R	S	R	R	R	S	R	S	S	S
D109	S	R	S	S	S	S	R	S	S	S	S	S	R	S	R	R	S	S	S	S	R	S
D131	S	R	S	S	S	S	R	S	S	S	S	S	R	S	R	R	S	S	S	S	R	S

S10. Patients with *S. odorifera infection and antibiogram*

Patient code	Antibiotics and susceptibility profile																			
	IMP	AMX	CAZ	FOX	CTX	CXM	COT	ERY	AMK	GEN	CIP	OFX	NAL	COL	PRL	TET	VAN	NIT	ATM	AMC	FOS
K291	S	S	S	S	S	S	S	S	S	S	S	S	S	S	R	S	S	S	S	S	S
K293	S	R	R	R	R	R	R	R	S	S	R	R	R	S	R	R	R	R	R	R	R
K294	S	R	R	R	R	R	R	R	S	S	R	R	R	S	R	R	R	R	R	R	R
